# Effect of High-Intensity Power Training on Cognitive Function in Older Adults With Type 2 Diabetes: Secondary Outcomes of the GREAT2DO Study

**DOI:** 10.1093/gerona/glac090

**Published:** 2022-04-18

**Authors:** Ren Ru Zhao, Yorgi Mavros, Jacinda Meiklejohn, Kylie A Anderberg, Nalin Singh, Shelley Kay, Michael K Baker, Yi Wang, Mike Climstein, Anthony O’Sullivan, Nathan De Vos, Bernhard T Baune, Steven N Blair, David Simar, Maria A Fiatarone Singh

**Affiliations:** Exercise Health and Performance Faculty Research Group, Faculty of Medicine and Health, University of Sydney, Camperdown, Sydney, New South Wales, Australia; Clinical Rehabilitation Centre, Faculty of Physical Education and Health Sciences, University of Longyan, Longyan, Fujian, China; Exercise Health and Performance Faculty Research Group, Faculty of Medicine and Health, University of Sydney, Camperdown, Sydney, New South Wales, Australia; Exercise Health and Performance Faculty Research Group, Faculty of Medicine and Health, University of Sydney, Camperdown, Sydney, New South Wales, Australia; Exercise Health and Performance Faculty Research Group, Faculty of Medicine and Health, University of Sydney, Camperdown, Sydney, New South Wales, Australia; Exercise Health and Performance Faculty Research Group, Faculty of Medicine and Health, University of Sydney, Camperdown, Sydney, New South Wales, Australia; Centre for Medical Psychology and Evidence Based Decision Making, Faculty of Medicine, University of Sydney, Camperdown, Sydney, New South Wales, Australia; Research Ethics and Integrity, Australian Catholic University, Strathfield, New South Wales, Australia; Clinical Exercise Physiology, School of Behavioural and Health Sciences, Australian Catholic University, Strathfield, New South Wales, Australia; Lipid Metabolism and Cardiometabolic Disease Laboratory, Baker Heart and Diabetes Institute, Melbourne, Victoria, Australia; School of Health and Human Sciences, Southern Cross University, Gold Coast, Queensland, Australia; Department of Endocrinology, Faculty of Medicine, University of New South Wales, Sydney, New South Wales, Australia; The Centre for STRONG Medicine, Balmain Hospital, Sydney, New South Wales, Australia; Department of Psychiatry, Melbourne Medical School, The University of Melbourne, Melbourne, Victoria, Australia; The Florey Institute of Neuroscience and Mental Health, The University of Melbourne, Melbourne, Victoria, Australia; Exercise Science Arnold School of Public Health, University of South Carolina, Columbia, South Carolina, USA; School of Medical Sciences, Faculty of Medicine, University of New South Wales, Sydney, New South Wales, Australia; Exercise Health and Performance Faculty Research Group, Faculty of Medicine and Health, University of Sydney, Camperdown, Sydney, New South Wales, Australia; Sydney Medical School, University of Sydney, Camperdown, Sydney, New South Wales, Australia

**Keywords:** Cognition, Power training, Type 2 diabetes

## Abstract

We sought to determine the effects of 12 months of power training on cognition, and whether improvements in body composition, muscle strength, and/or aerobic capacity (VO_2peak_) were associated with improvements in cognition in older adults with type 2 diabetes (T2D). Participants with T2D were randomized to power training or low-intensity sham exercise control condition, 3 days per week for 12 months. Cognitive outcomes included memory, attention/speed, executive function, and global cognition. Other relevant outcomes included VO_2peak_, strength, and whole body and regional body composition. One hundred and three adults with T2D (mean age 67.9 years; standard deviation [*SD*] 5.9; 50.5% women) were enrolled and analyzed. Unexpectedly, there was a nearly significant improvement in global cognition (*p* = .05) in the sham group relative to power training, although both groups improved over time (*p* < .01). There were significant interactions between group allocation and body composition or muscle strength in the models predicting cognitive changes. Therefore, after stratifying by group allocation, improvements in immediate memory were associated with increases in relative skeletal muscle mass (*r* = 0.38, *p* = .03), reductions in relative body fat (*r* = −0.40, *p* = .02), and increases in knee extension strength were directly related to changes in executive function (*r* = −0.41, *p* = .02) within the power training group. None of these relationships were present in the sham group (*p* > .05). Although power training did not significantly improve cognition compared to low-intensity exercise control, improvements in cognitive function in older adults were associated with hypothesized improvements in body composition and strength after power training.

## Background

Loss of skeletal muscle mass (SMM) and strength is common in older adults with type 2 diabetes (T2D) (1). Factors such as protein-energy malnutrition, biologic aging, hormonal dysregulation, inflammation, oxidative stress, and physical inactivity can all contribute markedly to the decline of muscle mass and strength and the accrual of fat mass (2), which are also observed in this cohort with dementia and T2D (3,4). Reductions in muscle quantity and quality in patients with T2D are associated with metabolic deficits, which are related to obesity and cardiovascular disease (5). Furthermore, it has recently been observed that impairments in muscle mass and function in T2D may be related to cognitive deficits as well. For example, in a cross-sectional study of 1 235 individuals with T2D in Singapore, those with lower leg muscle mass had greater cognitive impairment (1). Another cross-sectional study reported that community-dwelling older people with diabetes-related dementia had significantly lower upper extremity strength compared to those with Alzheimer’s dementia, with or without concomitant diabetes (4). Other aspects of body composition and exercise capacity have also been linked to cognition in various cohorts. For example, among 615 healthy participants aged 20–89 in the Baltimore Longitudinal Study on Aging followed prospectively, lower aerobic capacity (VO_2peak_) was predictive of accelerated rates of cognitive decline (6). Other epidemiological evidence also suggests that higher physical activity levels and VO_2peak_ are associated with preservation of cognitive function (7). The relationship between obesity and cognition, however, is more heterogeneous. For example, obesity in midlife is associated with long-term risk of poor cognitive function and cognitive decline in later life (8). In contrast, the association of late-life obesity and cognitive function is controversial, with studies showing a reverse, positive, or no association (9) between obesity and cognitive decline in late-life. Thus, the effect of adiposity on cognitive function in older adults remains unclear.

The relationships between exercise capacity, body composition, and cognition in older adults with T2D specifically, as well as the ability of exercise interventions to improve cognition in this cohort, and whether such improvements may be mediated by changes in either body composition or fitness adaptations are currently insufficiently studied. Cognitive functioning is associated with changes in muscle strength and muscle mass and fat mass in healthy older adults, but it is unknown if such relationships are present in those with T2D (10,11). Although less well studied than aerobic exercise, power training can also benefit cognition in older adults (12) but potential cognitive benefit from power training in T2D patients specifically remains unknown. In our recent comprehensive systematic review of both observational data and experimental trials (13), we identified only 6 studies (including 3 randomized controlled trials) addressing these questions directly. The limited data available suggested that aerobic exercise or lifestyle interventions may improve some aspects of cognition in older adults with T2D or impaired glucose tolerance, including executive function, delayed memory, and global cognitive scores, but the effects were inconsistent and require further study. Notably, *no study of the effect of isolated power training on cognition in T2D* was identified. With regards to mediators, exercise-induced improvements in insulin sensitivity and glucose levels were associated with the observed cognitive benefits in some studies, but there was insufficient evidence exploring potential relationships with other physiological adaptations such as fitness or body composition. Therefore, detailed analyses of the relationships between exercise-induced improvements in exercise capacity, metabolic control, body composition, and other metabolic parameters that may potentially mediate and/or moderate the cognitive adaptations are needed.

Thus, we investigated these relationships in prespecified secondary outcomes of the Graded Resistive Exercise And Type 2 Diabetes in Older Adults (GREAT2DO) trial (14). We hypothesized that 12 months of power training would address not only the metabolic deficits in T2D (the primary outcomes of the trial), but also any deficits in fitness (muscle strength and VO_2peak_), body composition (muscle mass and adiposity), which were reported previously (15), as well as cognitive function in this cohort (the focus of the current analyses). We chose a variant of resistance training called high intensity power training as the intervention, as it would theoretically most specifically address the abnormalities in skeletal muscle in this older age cohort (fast-twitch, or type II skeletal muscle atrophy), and potentially therefore, any cognitive benefits associated with anabolic adaptation. We also hypothesized that improvements in body composition (increases in muscle mass and reductions in adiposity), as well as increased muscle strength, and VO_2peak_ over 12 months would be independently associated with improvements in cognition.

## Method

### Study Design

The GREAT2DO trial was a double-blind randomized, sham exercise controlled clinical trial of power training in older adults with T2D and metabolic syndrome. The Royal Prince Alfred Human Research Ethics Committee approved the study (X04–0064). Written informed consent was obtained from all participants, and the study was registered with the Australia New Zealand Clinical Trials Registry (ANZCTR12606000436572). Concealed allocation of randomization group assignment was done via a computer-generated online random number generator (www.randomization.com), stratified for age, sex, and use of insulin using randomly permuted blocks. Group assignments were put in sequentially numbered opaque envelopes and handed to participants by the blinded research assessor at the completion of all baseline testing.

### Study Population and Eligibility Criteria

Participants were recruited to the single university study site via general practitioner referral, targeted mail-outs, advertisements in local newspapers and seniors’ magazines and from brochures distributed to local medical practitioners and pharmacies. Participants were recruited from August 2006 to December 2010 with the final 12-month assessment in December 2011, and 5-year follow up completed in 2016. Participants were 103 community-dwelling persons aged 60 or older (52 men and 51 women) and insufficiently active (no progressive resistance training; structured exercise ≤1/week; less than 150 minutes/week low- to moderate-intensity walking or other aerobic exercise). Participants could be treated with diet alone, oral medication or insulin, or combination at the time of enrollment. Participants had to have stable chronic diseases and be willing to commit to a 12-month exercise training program, 3 times per week. Exclusionary criteria (as detailed in Supplementary Material―Protocol Paper) included significant cognitive impairment (operationalized as inability to comprehend informed consent presented by the research assistance or evidence of functional impairment related to cognition noted on examination by the study physician, consistent with a diagnosis of dementia).

### Interventions

The complete intervention details have been published (14). All training was fully supervised by experienced research assistants (exercise physiologists) in a community gym or outpatient clinic of a hospital. The training frequency was 3 days per week for both groups. Participants were blinded to the investigators’ hypotheses as to which was the preferred intervention, as both were presented as potentially beneficial.

### Power Training

Power training was used, in which the concentric phase was completed as fast as possible, and the eccentric phase over 3 seconds, and was supervised at a ratio of 1 trainer to 3–4 participants. Participants completed 3 sets of 8 repetitions, 3 days per week, on 5 Keiser (Keiser Sports Health Fitness Ltd., Fresno, CA) pneumatic resistance machines (leg press, knee extension leg flexion, chest press, and seated row) and were progressed continuously throughout the 12-month intervention to maintain the intended high-intensity. This high-intensity training was implemented by conducting one-repetition maximum testing every 2 weeks to maintain intensity at approximately 80% of current strength across the entire 12 months. This was complemented by daily objective (trainer) and subjective (participant) assessment of perceived exertion targeting a range of 15–18 (Hard) on a modified Borg Scale (16), with daily adjustment of weights as needed to maintain this level of intensity.

### Sham Exercise Control

The supervised sham exercise was performed using the same machines (at different hours of the day) used for power training, but designed not to notably increase heart rate or enhance VO_2peak_ or strength or other physiological outcomes. The lowest weight possible on each machine was used, and no progression was included, but the same volume of training (3 sets of 8 repetitions, 3 days per week × 12 months) as in the power training group was utilized. The speed of contraction was 3 seconds concentric, 1–3 seconds eccentric, to minimize any adaptations due to velocity of movement or eccentric contractions.

### Adverse Events

Adverse events were defined a priori as any exacerbations of the underlying disease, or new onset musculoskeletal, cardiovascular, or metabolic abnormality attributed directly to study protocols. Monitoring of adverse events over 12 months was achieved by weekly questionnaire/interview with proxy information obtained whenever necessary.

### Outcomes

All outcomes were assessed by a blinded research assistant at a site remote from the training interventions, at baseline, 6 and 12 months. The assessments took place between 48 and 96 hours after the last training session at 6 and 12 months, to avoid any acute bout effects on cognitive performance.

### Cognitive Outcomes

Memory tests included the word list memory, word list recall, and word list recognition subtests of the Consortium to Establish a Registry for Alzheimer’s Disease (17). Attention/speed was assessed by the Trail Making Test Part A (Trails A) (18). Executive function was measured with the Trail Making Test Part B (Trails B) and the Trail Making Test B minus A (Trails B minus A) (18). The global cognitive function was evaluated using the Modified Mini-Mental State Examination (MMMSE) (19).

### Anthropometric Measurements

Morning fasting stretch stature (wall-mounted Holtain stadiometer; Holtain Limited, Crymych Pembs, UK) and naked weight (weight in gown [kg] minus weight of gown [kg]) were measured in triplicate to the nearest 0.1 cm and 0.01 kg, respectively. The body mass index was calculated using the formula weight/height^2^ (kg/m^2^). Waist circumference was measured with Lufkin steel tape (W606 PM), at the midpoint between the lower rib margin and the iliac crest, in triplicate to the nearest 0.5 cm.

### Measures of Body Composition

Whole-body measures of body composition (SMM and body fat mass [BFM]) were determined using bioelectrical impedance analysis (BIA; RJL Systems, Clinton, MI) (20,21). Computed tomography (GE High-Speed CTI Scanner; Milwaukee, WI), used at the Royal Prince Alfred Hospital, Sydney, Australia, and was used to quantify visceral adipose tissue (VAT; cm^2^), mid-thigh muscle cross-sectional area (CSA; cm^2^), and muscle density (an index of intramyocellular lipid content assessed via mid-thigh muscle attenuation).

### Assessment of Aerobic Capacity

VO_2peak_ was determined using indirect calorimetry during a physician-administered, graded treadmill walking test with electrocardiographic monitoring to volitional fatigue.

### Assessment of Peak Strength

Testing was performed on pneumatic resistance machines (Keiser Sports Health Equipment Ltd). Participants’ one-repetition maximum determined on the knee extension machine was used for analyses in relation to cognition, as representative of major lower extremity muscle function.

### Statistical Analysis

An intention-to-treat analytic strategy was employed, with all randomized participants included regardless of dropout or discontinuation. Data were inspected visually and statistically for normality prior to use of parametric statistics. Data were represented as mean (standard deviation [*SD*]), mean (95% confidence intervals), median (interquartile range), or frequencies as appropriate. Non-normally distributed data were log-transformed for use with parametric statistics where possible.

A repeated measures linear mixed-effects model with first auto-regressive covariance structure was used to determine changes over time, using all available data without imputation, allowing for inclusion of all participants regardless of any missing follow-up data. Seven base models were initially created, each including one of the cognitive outcomes―word list memory, word list recall, word list recognition, Trails B minus A, Trails B, Trails A, and MMMSE. In each model, the cognitive outcome was entered as the dependent variable with age, sex, insulin usage at baseline (randomization stratification variables), education level, group, time, and group × time interactions entered as fixed effects, in addition to random effects added as an intercept and at the participant level. All covariates were selected a priori based on known associations with cognition in older adults or T2D (age, sex, and education), and/or because they were stratification factors in the randomization procedure (age, sex, and insulin usage). In addition, characteristics which were potentially related to the cognitive variables of interest and different by potentially clinically meaningful amounts were considered as additional covariates/confounders in a second set of models.

A multiple linear regression model was constructed to determine the independent contribution of change in body composition, VO_2peak_, or muscular performance to cognitive performance at trial completion―all variables identified a priori as likely to contribute to the adaptation of cognition following the intervention. Change in cognition (defined as cognitive score at 12 months minus cognitive score at baseline) was entered as the dependent variable. Age, sex, education, and insulin usage at baseline and the change in body composition/fitness variable, group, group × change in body composition/fitness variable entered as independent variables in the first set of models, and then any other relevant characteristics different between groups, as noted earlier. Separate multiple linear regression models were then constructed for each cognition variable to determine whether the association differed between groups by adding a group × variable of interest interaction term to each model. For any interactions that were significant (*p* < .05), the within-group associations were then explored using multiple linear regression models stratified by group and adjusted for age, sex, education, and insulin usage at baseline. Hedges’ bias corrected effect sizes (ES) were calculated for all outcomes. Statistical significance was assumed at the 0.05 level as all hypotheses were specified a priori, and all outcomes were secondary exploratory analyses in the overall trial. All data were analyzed using SPSS version 22 (IBM Corp., Armonk, NY).

## Results

### Recruitment and Participant Characteristics

**Figure 1.**
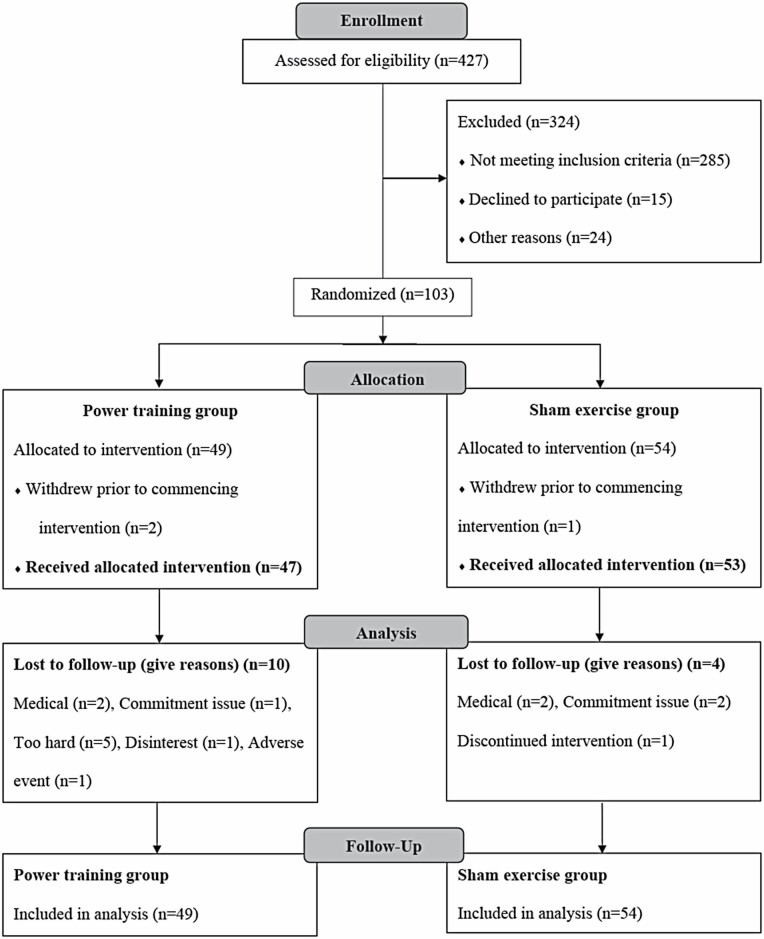
CONSORT participant enrollment flow diagram.

Figure 1 shows 427 people were assessed for eligibility. After telephone screening, the majority did not meet study criteria (*n* = 285) or declined to participate (*n* = 15), and a further 24 participants were either unable to commit or had medical contraindications. A total of 103 people agreed to participate, met the study criteria, and were randomized to either the power training (*n* = 49) or the sham group (*n* = 54). Three participants withdrew before commencing the intervention but were included in the analyses. Therefore, 100 began the intervention, 86 (37 power training and 49 sham) completed their 12-month follow-up, yielding a dropout rate after starting the intervention of 14%, due to: commitment (3%), dissatisfaction (1%), adverse event (1%), perception that it was too hard (5%), or medical issues (4%). Three percent (*n* = 3) discontinued training but had follow-up testing, all because of commitment issues, not health or adverse events.

Baseline characteristics of participants are shown in Table 1. Participants in the sham group had a lower MMMSE score and higher levels of HbA1c, older age, longer duration of T2D and have better average SF-36 score for a mental component summary but appeared similar in other demographic, body composition, and metabolic health parameters. The most common chronic conditions were coronary artery disease (19%), depression (36%), hypertension (74%), dyslipidemia (44%), osteoarthritis (66%), and peripheral vascular disease (20%).

**Table 1.** Baseline Participant Characteristics

	Total	Power Training Group	Sham Exercise Group
	*n* = 103	*n* = 49	*n* = 54
*Demographics*			
Age (years)	67.9 ± 5.5	66.9 ± 4.7	68.8 ± 6.1
Women sex (%)	52.0 (50.5)	25.0 (51.0)	27.0 (50.0)
Educational level (years)	13.6 ± 3.7	13.5 ± 3.7	13.7 ± 3.7
Ethnic origin			
Caucasian (%)	99.0 (96.1)	49.0 (100.0)	50.0 (92.6)
Asian (%)	2.0 (1.9)	0.0 (0.0)	2.0 (3.7)
Indian (%)	2.0 (1.9)	0.0 (0.0)	2.0 (3.7)
*Medical history*			
History of smoking (%)	39.0 (38.0)	18.0 (37.0)	21.0 (39.0)
Current smoker (%)	6.0 (6.0)	3.0 (6.0)	3.0 (6.0)
Duration of diabetes (years)	7.0 (1.0, 29.0)	6.5 (1.0, 28.0)	7.5 (1.0, 29.0)
Number of chronic diseases (*n*)	5.1 ± 1.9	5.1 ± 1.9	5.0 ± 1.8
Stroke (%)	3.0 (3.0)	0.0 (0.0)	0.0 (0.0)
Cardiovascular disease (%)	50.0 (49.0)	23.0 (16.0)	27.0 (50.0)
Myocardial infarction/angina (%)	20.0 (19.0)	8.0 (50.0)	12.0 (22.0)
Previous depression or GDS score >10/30*	37.0 (36.0)	20.0 (41.0)	17.0 (32.0)
Hypertension (%)	76.0 (74.0)	37.0 (76.0)	39.0 (72.0)
Dyslipidemia (%)	45.0 (44.0)	24.0 (49.0)	21.0 (39.0)
Osteoarthritis (%)	68.0 (66.0)	33.0 (67.0)	35.0 (65.0)
Peripheral vascular disease (%)	21.0 (20.0)	10.0 (20.0)	11.0 (20.0)
Systolic blood pressure (mm Hg)	128.0 ± 13.0	128.0 ± 12.0	128.0 ± 14.0
Diastolic blood pressure (mm Hg)	70.0 ± 7.0	70.0 ± 7.0	69.0 ± 7.0
Total number of medications/day (*n*)	5.7 ± 3.0	5.2 ± 2.8	6.1 ± 3.1
*Metabolic health*			
Serum glucose, fasting (mmol/L)	6.5 (4.2, 17.6)	6.4 (4.6, 17.6)	6.6 (4.2, 16.4)
HbA1c (%)	7.1 ± 1.1	6.9 ± 0.9	7.3 ± 1.2
HOMA2-IR	2.7 ± 1.2	2.6 ± 1.0	2.9 ± 1.3
Insulin users (%)	16.0 (10.0)	7.0 (14.0)	9.0 (17.0)
Metformin users (%)	74.0 (72.0)	36.0 (73.0)	38.0 (70.0)
Metformin dosage (mg/day)	1 544.0 ± 659.0	1 526.0 ± 619.0	1 562.0 ± 703.0
C-reactive protein (mmol/L)	2.8 (0.0, 21.0)	3.6 (0.0, 13.0)	2.8 (0.0, 21.0)
Total cholesterol (mmol/L)	4.2 (2.2, 7.9)	4.2 (3.3, 7.4)	4.2 (2.2, 7.9)
Triglycerides (mmol/L)	1.5 (0.5, 3.9)	1.5 (0.5, 3.1)	1.5 (0.5, 3.9)
High-density lipoprotein (mmol/L)	1.2 ± 0.3	1.2 ± 0.3	1.2 ± 0.4
Low-density lipoprotein (mmol/L)	2.2 (1.0, 6.0)	2.2 (1.0, 5.0)	2.0 (1.0, 6.0)
*Body composition*			
Body mass index (kg/m^2^)	31.6 ± 5.4	31.5 ± 4.7	31.6 ± 6.0
Body mass (kg)	89.1 ± 17.1	89.5 ± 15.2	88.7 ± 18.8
Total abdominal adipose tissue (cm^2^)	421.6 ± 117.5	431.3 ± 110.8	412.6 ± 123.8
Visceral adipose tissue (cm^2^)	216.9 ± 89.2	222.8 ± 84.8	208.2 ± 93.3
Subcutaneous abdominal adipose tissue (cm^2^)	206.6 ± 90.4	208.9 ± 93.0	204.4 ± 88.8
Mid-thigh muscle cross-sectional area (cm^2^)	109.3 ± 24.1	110.8 ± 26.3	108.0 ± 22.1
Mid-thigh muscle attenuation^†^	84.1 ± 2.3	84.1 ± 2.2	84.1 ± 2.3
Skeletal muscle mass (kg)	26.6 ± 6.6	27.0 ± 7.1	26.2 ± 6.1
Fat-free mass (kg)	56.1 ± 10.6	56.7 ± 11.3	55.5 ± 10.1
Total body fat mass (kg)	32.7 ± 11.7	32.7 ± 10.1	32.7 ± 13.1
*Physical and mental health summary scales*			
SF-36 Mental component summary	51.1 (0.0, 65.0)	50.6 (0.0, 60.0)	52.3 (27.0, 65.0)
SF-36 Physical component summary	46.3 (0.0, 62.0)	47.3 (0.0, 62.0)	45.7 (18.0, 60.0)
* Cognitive function*			
Modified Mini-Mental State Examination (0–100)	96.0 (82.0, 100.0)	96.5 (85.0, 100.0)	94.0 (82.0, 100.0)
Trail Making Test Part A (s)^‡^	40.6 ± 12.6	40.1 ± 12.0	40.8 ± 13.2
Trail Making Test Part B (s)^‡^	84.7 (45.3, 300.0)	82.7 (50.6, 241.0)	85.5 (45.3, 300.0)
Trail Making Test Part B minus Trail Making Test Part A (s)^‡^^,^^§^	45.2 (−2.2, 267.1)	42.7 (−2.2, 264.9)	47.5 (12.5, 221.4)
Word list memory (*n*)	21.3 ± 3.8	21.1 ± 3.4	21.1 ± 4.2
Word list recall (*n*)	7.0 ± 1.8	6.9 ± 1.4	7.1 ± 2.1
Word list recognition (*n*)	10.0 (6.0, 10.0)	10.0 (6.0, 10.0)	10.0 (8.0, 10.0)

*Notes:* GDS = Geriatric Depression Scale; HbA1c = Glycated Hemoglobin; HOMA2-IR = Homeostatic Model of Assessment 2 of Insulin Resistance; *SD* = standard deviation. Normally distributed data presented as mean ± *SD*. Non-normally distributed data were presented as median (range) or frequency (%) as appropriate.

*Depression was defined as either a history of depression or a score of 10 higher on the GDS.

^†^Thigh muscle attenuation is a unitless measure of intramyocellular lipid where higher values indicate greater lipid levels.

^‡^Lower score indicates better function. For all other tests, higher score indicates better function.

^§^Trail Making Test Part B minus Trail Making Test Part A score is calculated as the difference score between Trail Making Test B and Trail Making Test A times in seconds (s).

### Training Adherence

Total weeks of exercise were equivalent between power training and sham (mean [*SD*] 53 [3] vs 54 [12]; *p* = .73). Median (interquartile) compliance to the intervention was 82% (16%), median attendance rate was not significantly different in the power training group compared to sham (76% [15%] vs 83% [15%]; *p* = .09).

### Adverse Events

There were 8 adverse events over 12 months attributed to the interventions: 7 in the power training (3 syncopal episodes, 1 hamstring strain, 1 back pain, 1 subscapularis tear, and 1 tear of rotator cuff muscle) and 1 in the sham group (1 exacerbation of preexisting umbilical hernia).

### Outcomes

#### Changes in cognitive function

There were no significant effects of group assignment on cognitive function over the 12 months (no group × time interactions). Unexpectedly, there was a trend for greater increases in MMMSE in the sham group relative to power training (Tables 2 and 3; *p* = .05; moderate ES of 0.52). On the other hand, there were significant improvements in Trails B, Trails A, word list memory, and word list recall (*p* < .001) over time, without regard to group assignment. There were no time or group × time interactions observed for either word list recognition and Trails B minus A. Similarly, the results remained unchanged in an additional model after adjustment for age, sex, educational level, insulin usage, duration of diabetes, HbA1c, and MMMSE at baseline (Supplementary Table 1).

**Table 2.** Scores for Primary Outcomes at All Time Points for Each Randomization Group

Cognitive Outcome	Power Training Group	Sham Exercise Group
*Modified Mini-Mental State*		
*Examination* (0–100)		
Baseline	96.50 (86.00, 100.00)	94.00 (82.00, 100.00)
6 months	97.00 (84.00, 100.00)	96.00 (78.00, 100.00)
12 months	7.00 (89.00, 100.00)	98.00 (79.00, 100.00)
Trail Making Test Part A (s)*		
Baseline	41.16 ± 12.32	40.24 ± 12.83
6 months	40.76 ± 11.64	40.46 ± 11.58
12 months	36.55 ± 11.11	34.38 ± 10.37
Trail Making Test Part B (s)*		
Baseline	82.03 (50.63, 241.00)	85.91 (45.25, 300.00)
6 months	86.63 (51.20, 213.00)	83.24 (41.78, 185.94)
12 months	81.48 (42.31, 219.40)	73.80 (41.38, 199.69)
*Trail Making Test Part B minus*		
Trail Making Test Part A (s)*		
Baseline	41.37 (-2.18, 190.59)	47.47 (13.64, 221.38)
6 months	48.16 (15.51, 169.66)	46.29 (-11.33, 147.10)
12 months	48.01 (5.81, 184.87)	43.17 (5.18, 154.06)
Word list memory (0–30)		
Baseline	21.30 ± 3.44	21.20 ± 3.43
6 months	22.68 ± 3.36	23.28 ± 3.38
12 months	24.12 ± 3.29	24.15 ± 3.35
Word list recall (0–10)		
Baseline	6.88 ± 1.68	7.16 ± 1.67
6 months	7.44 ± 1.63	7.65 ± 1.64
12 months	8.21 ± 1.59	7.95 ± 1.63
Word list recognition (0–10)		
Baseline	10.00 (6.00, 10.00)	10.00 (8.00, 10.00)
6 months	10.00 (6.00, 10.00)	10.00 (6.00, 10.00)
12 months	10.00 (9.00, 10.00)	10.00 (7.00, 10.00)

*Notes: n* = 103 for all outcomes. All data were normally distributed and raw data used except for Modified Mini-Mental State Examination, Trail Making Test Part B minus Trail Making Test Part A, word list recognition, and Trail Making Test Part B. Trail Making Test Part B data were transformed by taking the inverse before use with parametric statistics. Modified Mini-Mental State Examination, Trail Making Test Part B minus Trail Making Test Part A, and word list recognition data required log-transformed before use with parametric statistics. Data for Modified Mini-Mental State Examination, Trail Making Test Part B minus Trail Making Test Part A, and word list recognition data presented as median (range) of raw values. Data for Trail Making Test Part A, word list memory, and word list recall represented the estimated marginal means ± standard deviation from repeated measures linear mixed models including all 3 time points, with fixed effects of time, group, and their interactions, and adjusted for age, sex, highest level of education, and insulin user.

*Lower score indicates better function. For all other assessment, a higher score indicates better function.

**Table 3.** Repeated Measures Linear Mixed Model Analysis and Effect Sizes for All Cognitive Outcomes

Statistical Model	Time, *p* Value	Group × Time, *p* Value	Relative Effect Size (95% CI)
*Modified Mini-Mental State*			
*Examination*			
Model (0, 6, 12)	<.001	.05	−0.52 (−1.04, 0.01)
Trail Making Test Part A			
Model (0, 6, 12)	<.001	.75	0.10 (−0.42, 0.62)
Trail Making Test Part B			
Model (0, 6, 12)	<.02	.12	0.16 (−0.37, 0.68)
*Trail Making Test Part B minus*			
Trail Making Test Part A			
Model (0, 6, 12)	.89	.25	0.31 (−0.22, 0.83)
Word list memory			
Model (0, 6, 12)	<.001	.54	−0.04 (−0.56, 0.48)
Word list recall			
Model (0, 6, 12)	<.001	.20	0.32 (−0.21, 0.84)
Word list recognition			
Model (0, 6, 12)	.25	.39	0.00 (−0.52, 0.52)

*Notes:* CIs = confidence intervals. *n* =103 for all outcomes. All data were normally distributed and raw data used except for Modified Mini-Mental State Examination, Trail Making Test Part B minus Trail Making Test Part A, word list recognition, and Trail Making Test Part B. Trail Making Test Part B data were transformed by taking the inverse before use with parametric statistics. Modified Mini-Mental State Examination, Trail Making Test Part B minus Trail Making Test Part A, and word list recognition data required log-transformed before use with parametric statistics. A separate linear mixed.

### Association Between Changes in Cognitive Function and Changes in Body Composition

Given the lack of any significant effects of group assignment on cognition, associations with other outcomes were explored across the combined cohort. Increases in relative SMM and reductions in relative body fat were associated with improvements in word list memory (*r* = 0.63, *p* = .04 and *r* = −0.73, *p* = .02, respectively), as hypothesized. Increases in absolute SMM also tended to be associated with improvements in word list memory and word list recall (*r* = 0.62, *p* = .09 and *r* = 0.60, *p* = .09, respectively). Similarly, reductions in BFM tended to be associated with improvements in word list memory (*r* = −0.47, *p* = .11). Changes in cognitive outcomes were not associated with changes in VAT, body mass index, waist circumference, or thigh muscle CSA (Supplementary Table 2; *p* > .05). As noted in Supplementary Table 4, a significant association between changes in relative SMM and relative BFM and changes in word list memory persisted even after adjustment for age, sex, educational level, insulin usage, duration of diabetes, HbA1c, and MMMSE at baseline.

**Figure 2.**
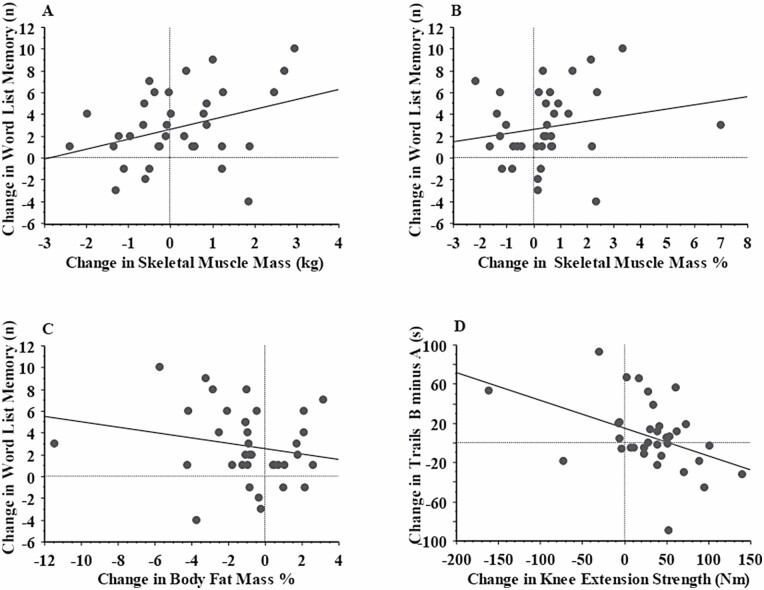
Association between changes in body composition and knee extension strength and changes in memory and executive function in the power training group. (A) Changes in memory versus changes in skeletal muscle mass; standardized coefficient (*r*) = 0.37, *p* < .05; (B) Changes in memory versus changes in percent skeletal muscle mass; *r* = 0.38, *p* = .03; (C) Changes in memory versus changes in percent body fat mass; *r* = −0.40, *p* = .02; and (D) Changes in executive function test Trails B minus A versus changes in knee extension strength; *r* = −0.41, *p* = .02.

There were significant group × relative SMM/relative body fat interactions in these models, suggesting that the relationships were altered by group assignment, and therefore stratified analyses were conducted, as specified a priori. After stratifying by exposure to power training or sham, the associations between improvements in SMM and word list memory (Figure 2A; *r* = 0.37, *p* < .05, respectively) were strengthened in those who received power training. Additionally, improvements in relative SMM and relative body fat were now significantly associated with increases in word list memory score (Figure 2B and C; *r* = 0.38, *p* = .03 and *r* = −0.40, *p* = .02, respectively). By contrast, no associations between body composition and any cognitive changes were observed in individuals randomized to receive sham (Supplementary Table 2; *p* > .05).

### Association Between Changes in Cognitive Function and Exercise Capacity

Results are shown in Supplementary Table 3. Improvements in muscle strength were associated with improvement in executive function via the Trails B test minus Trails A test (*r* = −0.93, *p* < .01). There were group × strength interactions, and therefore we stratified the regression models. Increases in knee extension strength were associated with improvements in Trails B minus A in the power training group (Figure 2D; *r* = −0.41, *p* = .02) but not in sham group (*r* = 0.10, *p* = .52). Changes in relative and absolute VO_2peak_ were not associated with any changes in cognitive function in the whole cohort (*p* > .05). These associations remained significant after adjustment for all covariates (Supplementary Tables 4 and 5).

## Discussion

These outcomes of the GREAT2DO trial did not support our hypothesis that power training would benefit cognition compared to the sham control condition in older adults with T2D after 12 months of training. However, both groups exhibited improvements in most aspects of cognition over time. As hypothesized, increases in muscle mass and reductions in adiposity were related to improvements in memory in the power training group, without such relationships evident in the sham exercise group. Also as hypothesized, increased muscle strength was associated with improved cognition (executive function), in the power training but not the sham group, whereas VO_2peak_ change was unrelated to cognitive outcomes.

Contrary to our hypothesis, the power training group tended to perform more poorly compared with the sham group in global cognitive function at 12 months, although both groups improved over time. This paradoxical effect of power training result is inconsistent with the findings reported in previous studies of power training in older adults (22,23). For example, in the Study of Mental and Resistance Training (SMART), 6 months of high-intensity power training improved cognition significantly across 18 months of follow-up in older adults with mild cognitive impairment (24). The precise reasons for this significant blunting power training benefit compared with sham group is unclear. Most importantly, baseline cognition was normal in GREAT2DO, and baseline MMMSE values in the power training group were higher than in the sham group. This may have limited the ability to increase cognitive scores in this group and minimized the difference between groups. The GREAT2DO cohort, with a mean age of 68 years at the time of cognitive assessment, may have just begun to enter the time of greatest susceptibility to cognitive deficits, and thus our sensitivity to detect changes may have been insufficient, in contrast to the SMART study (24). Second, we did not have an untreated control group, in order to maintain the double-blind design of the trial. It is possible that a usual care control group may have experienced a stabilization or decline in cognition compared to our two exercise groups, potentially magnifying either time or group × time effects. Third, the sham exercise group had a significant change to their lifestyle, including 3 days a week of travel to an exercise gym, and 30–40 minutes of fully supervised novel exercise using resistance training equipment 3 days per week for 12 months, including social interaction with the trainer and the other participants in the gym setting. This active transport and socialization may have increased the potential of the low-intensity exercise group to benefit them cognitively, despite the intent to minimize any physiological adaptations with this paradigm. Finally, some of the power training participants may not have been able to adhere consistently to the high-intensity repetitions prescribed due to underlying musculoskeletal issues, thus further minimizing the intended difference in intensity between groups. Our intent was to make the 2 regimens and experiences similar in every way *other than intensity,* in order to isolate the hypothesized mechanism of the exercise benefit. However, it may be that other aspects of the exercise and experimental setting may have contributed to some of the observed cognitive benefits. Given that older adults with T2D are at known high risk of cognitive decline, the fact that participation in both groups resulted in *improvements* in cognition after 12 months is clinically important and deserves additional study. Few other options exist to prevent cognitive decline and dementia in this cohort.

### Changes in Skeletal Muscle and Cognition

As hypothesized, increases in SMM were significantly associated with improvements in memory following high-intensity power training. To the best of our knowledge, this is the first study to examine the association between improvements in SMM and cognition after power training in older adults with T2D. The lack of relationship in the sham group could be due to the fact that any increases in SMM not due to power training could represent weight gain from dietary intake rather than anabolic adaptations, which may have less physiological benefits. In contrast to our findings, a previous study in healthy older women showed no relationship between changes in lean body mass and changes in cognition following 12 months of power training (11). This could be due to the imprecision of dual-energy absorptiometry for the assessment of SMM, or perhaps to the closer relationship between body composition and muscle function and cognition in T2D compared to other cohorts. Supporting this relationship, we also showed that muscle strength changes were related to cognitive benefit (executive function), and again only in the power training group. By contrast, VO_2peak_ changes did not predict cognitive changes. These outcomes are similar to our reported findings in the SMART cohort with mild cognitive impairment, in whom changes in muscle strength were shown to mediate 63% of the improvement in global cognition, whereas VO_2peak_ at baseline or over time did not predict cognitive benefit (25).

Thus, the current study provides novel data supporting the potential mechanistic or moderating effects of factors underlying musculoskeletal adaptations on cognition in older adults with T2D. Exploration of potential linkages between SMM and brain adaptations to power training, such as an increase in anabolic hormones (Insulin-like growth factor-1 [IGF-1] and other myokines) or down regulation of inflammatory cytokines is required to advance understanding in this field. The fact that SMM changes had no relationship to cognitive changes in the sham exercise group supports the suggestion that other psychosocial aspects of enrollment in the trial may underlie the benefits in these participants. This supports the need for very careful design of such trials, and the limited inferences that can be drawn from uncontrolled or unblinded studies.

Given the novelty of these findings, future studies are needed directed toward the identification of the causal pathways linking adaptations in SMM and cognitive function after power training. Published data support the clinical relevance of these relationships. In individuals with T2D, the inability to preserve muscle mass is attributed to glycemic control, insulin resistance, and adipose tissue deposition in skeletal muscles (26), and thus it is possible that increases in SMM after power training simply increased the available storage depot for glucose in our study (15), which could ultimately lead to improved brain plasticity and cognition (27). Furthermore, cognitive impairment is associated with systemic inflammatory abnormalities that are also implicated in sarcopenia (28). Although our measures of inflammatory processes are limited in this study, we have reported an independent relationship between SMM and insulin in this study (*p* < .05) (15), an anabolic hormone that may have neurotrophic and neuroprotective properties. A previous study has reported that insulin levels are associated with better cognition in early Alzheimer’s disease (29). Muscle is our largest metabolically active organ, thus improving SMM through anabolic exercise may improve the metabolic health of older adults with T2D, and thereby cognition. IGF-1 and brain-derived neurotrophic factor (BDNF) deficiency have been linked to cognitive decline and dementia in older adults (30), whereas older adults who underwent a 24-week physical activity intervention experienced an increase in IGF-1 concomitant with improvements in cognitive function (23). Improvements in BDNF have been found to mediate the effects of a 12-month physical activity intervention on executive function in adults (31), consistent with previously reported animal data linking exercise, BDNF, and neuroplasticity (32).

### Changes in Adiposity and Cognition

Our linear regression models indicated that reductions in relative adiposity following power training were associated with improvements in cognition only in the power training group. Similarly, a reduction in BFM after 12 months of power training in older women was associated with enhanced memory (11). These authors concluded that adiposity might contribute to cognitive impairment. Furthermore, our result is supported by biochemical studies demonstrating the effects of adipose metabolite secretion on brain health (33). Critically, current evidence suggests that adipose tissue is a major contributor to circulating inflammatory markers such as C-reactive protein (CRP) and interleukin-6 (IL-6), and may underline the association between adiposity and cognitive decline and dementia (34). Secretion of CRP and IL-6 within subcutaneous and VAT has also been reported (35,36). Thus, reductions in circulating CRP and IL-6 can be mediated both directly and indirectly, by reductions in adipose tissue. The finding that CRP and IL-6 are produced by both subcutaneous and VAT supports our finding that reductions in BFM (inclusive of both depots) predicted improvements in cognition in our cohort, rather than VAT in isolation. Thus, methods to optimize reduced adiposity after power training (such as combinations with dietary interventions) should be investigated as a way to reduce systemic inflammation and thus maximize improvements in cognitive function.

### Changes in Strength and Cognition

Our finding of a significant independent association between increased lower body strength and improved cognitive performance concurs with the results of a previous study (25) examining the effects of strength changes on cognition. These authors found a positive impact of greater strength gains on measures of executive function and global cognition after 12 months of power training in the SMART cohort with mild cognitive impairment. Similarly, another study showed that after power training, but not multicomponent training over 3 months, increases in strength were significantly associated with improved executive function in healthy older adults (37). Thus, the current study’s empirical data substantively extend epidemiological literature linking strength with the rate of cognitive decline and incident dementia (4). To the best of our knowledge, there are no other studies of persons with diabetes that have demonstrated the link between improvements in strength and cognition after power training. Thus, the current study provides novel data suggesting the need to investigate potential physiological factors underlying strength adaptations which may also benefit cognition after power training in older adults with T2D.

### Changes in Aerobic Capacity and Cognition

No associations were observed between changes in VO_2peak_ and improvements in cognitive function. Although there is accumulating evidence from cross-sectional and prospective cohort studies that cardiorespiratory is associated with enhanced cognitive function (6,38), Erickson conducted the only experimental trial that included changes in measures of cognitive function and aerobic fitness, as measured by VO_2peak_, after aerobic training, and found no significant associations between change in aerobic fitness and cognitive performance after 1 year of aerobic training in healthy older adults (39), suggesting that increased hippocampal volume may be attributed to improved memory through different pathways such as increased BDNF (31). Furthermore, a study of aerobic exercise in adults with impaired glucose tolerance (40) did not report whether changes in VO_2peak_ were related to executive function change, even though it was shown to improve. Finally, in the SMART study of mild cognitive impairment, although power training improved VO_2peak_ by 8%, this change was unrelated to cognitive benefits of training (25). Thus, the current findings support and extend existing experimental literature suggesting that improvements in VO_2peak_ do not mediate improvements in cognition after either aerobic or resistance training in older adults, in contrast to the many epidemiological investigations documenting the association between cardiorespiratory fitness and cognition (41). This could be due to the known significant contribution of genetics to VO_2peak_, or to the many other lifestyle and physiological characteristics associated with having a high VO_2peak_ in observational and experimental studies, all of which may be linked to risk of cognitive decline.

### Limitations

The limitations of our study include a lack of a control group without exercise, and the fact that the exercise program in the sham group may have provided unintentionally potent benefits due to active transport and social contact required, as well as potentially an unanticipated effect of low-intensity muscle pump/contractions on cognitive processes. The cognitive tests may not have included all aspects of cognition in the neurocognitive battery chosen. Finally, the presence of a sex effect on the cognitive adaptation to exercise is possible. However, the current investigation was not powered to warrant examination of any sex-specific adaptations.

## Conclusion

A 12-month, high-intensity power training program had no superior effects on cognitive function in older adults with T2D compared with a low-intensity, sham exercise condition. However, cognition improved in both groups over time, which is unexpected in an older cohort with T2D and multiple comorbidities. Notably, increases in SMM and strength and reductions in adiposity achieved through power training were all associated with improved cognition, whereas no such relationships were seen in the sham control group. Further large-scale trials are warranted to confirm and extend our findings, investigate the underlying mechanisms linking body composition and metabolism with brain morphology and neuroplasticity, and continue to explore the potential value of exercise targeted towards robust muscle strength and body composition adaptations to combat cognitive decline and dementia risk in this vulnerable cohort with T2D.

## Supplementary Material

glac090_suppl_Supplementary_Material

## Data Availability

Data are stored securely in a University server and the authors can be contacted regarding access if needed. Other analyses and data entry into this data set are still in progress so the data are not available in the public domain yet.

## Notes

**Clinical Trials Registration Number:** ANZCTR12606000436572
