# Supplementary material for: Effect of High-Intensity Power Training on Cognitive Function in Older Adults With Type 2 Diabetes: Secondary Outcomes of the GREAT2DO Study
Source: J Gerontol A Biol Sci Med Sci. 2022 Apr 18;77(10):1975–85. doi: 10.1093/gerona/glac090 (PMC9536451; doi:10.1093/gerona/glac090)
Supplement: glac090_suppl_Supplementary_Material [file glac090_suppl_supplementary_material.pdf]

CONSORT 2010 checklist of information to include when reporting a randomised trial*

Section/Topic	Item No	Checklist item	Reported on page No
**Title and abstract**			
	1a	Identification as a randomised trial in the title	Supplementary File 2 Page 1
	1b	Structured summary of trial design, methods, results, and conclusions (for specific guidance see CONSORT for abstracts)	Supplementary File 2 Page 1
**Introduction**			
Background and objectives	2a	Scientific background and explanation of rationale	Supplementary File 2 Page 2
	2b	Specific objectives or hypotheses	Supplementary File 2 Page 2
**Methods**			
Trial design	3a	Description of trial design (such as parallel, factorial) including allocation ratio	Supplementary File 2 Page 7
	3b	Important changes to methods after trial commencement (such as eligibility criteria), with reasons	There were no changes
Participants	4a	Eligibility criteria for participants	Supplementary File 2 Page 3
	4b	Settings and locations where the data were collected	Supplementary File 2 Page 5
Interventions	5	The interventions for each group with sufficient details to allow replication, including how and when they were actually administered	Supplementary File 2 Page 5
Outcomes	6a	Completely defined pre-specified primary and secondary outcome measures, including how and when they were assessed	Supplementary File 2 Page 5
	6b	Any changes to trial outcomes after the trial commenced, with reasons	There were no changes
Sample size	7a	How sample size was determined	Supplementary File 2 Page 3
	7b	When applicable, explanation of any interim analyses and stopping guidelines	There were no

			changes
Randomisation:			
Sequence generation	8a	Method used to generate the random allocation sequence	Supplementary File 2 Page 7
	8b	Type of randomisation; details of any restriction (such as blocking and block size)	Supplementary File 2 Page 7
Allocation concealment mechanism	9	Mechanism used to implement the random allocation sequence (such as sequentially numbered containers), describing any steps taken to conceal the sequence until interventions were assigned	Supplementary File 2 Page 7
Implementation	10	Who generated the random allocation sequence, who enrolled participants, and who assigned participants to interventions	Supplementary File 2 Page 7
Blinding	11a	If done, who was blinded after assignment to interventions (for example, participants, care providers, those assessing outcomes) and how	Supplementary File 2 Page 8
	11b	If relevant, description of the similarity of interventions	Supplementary File 2 Page 5
Statistical methods	12a	Statistical methods used to compare groups for primary and secondary outcomes	Supplementary File 2 Page 8
	12b	Methods for additional analyses, such as subgroup analyses and adjusted analyses	Supplementary File 2 Page 8
**Results**			
Participant flow (a diagram is strongly recommended)	13a	For each group, the numbers of participants who were randomly assigned, received intended treatment, and were analysed for the primary outcome	Supplementary File 2 Page 10
	13b	For each group, losses and exclusions after randomisation, together with reasons	Supplementary File 2 Page 9
Recruitment	14a	Dates defining the periods of recruitment and follow-up	Supplementary File 2 Page 9
	14b	Why the trial ended or was stopped	Supplementary File 2 Page 9
Baseline data	15	A table showing baseline demographic and clinical characteristics for each group	Supplementary File 2 Page 9
Numbers analysed	16	For each group, number of participants (denominator) included in each analysis and whether the analysis was by original assigned groups	Supplementary File 2 Page 9
Outcomes and	17a	For each primary and secondary outcome, results for each group, and the estimated effect size and its precision	Supplementary

estimation		(such as 95% confidence interval)	File 2 Page 9
	17b	For binary outcomes, presentation of both absolute and relative effect sizes is recommended	Supplementary File 2 Page 9
Ancillary analyses	18	Results of any other analyses performed, including subgroup analyses and adjusted analyses, distinguishing pre-specified from exploratory	Supplementary File 2 Page 9
Harms	19	All important harms or unintended effects in each group (for specific guidance see CONSORT for harms)	Supplementary File 4 Page 9
**Discussion**			
Limitations	20	Trial limitations, addressing sources of potential bias, imprecision, and, if relevant, multiplicity of analyses	Supplementary File 2 Page 10 and 12
Generalisability	21	Generalisability (external validity, applicability) of the trial findings	Supplementary File 2 Page 10 and 12
Interpretation	22	Interpretation consistent with results, balancing benefits and harms, and considering other relevant evidence	Supplementary File 2 Page 10 and 12
**Other information**			
Registration	23	Registration number and name of trial registry	Supplementary File 2 Page 2
Protocol	24	Where the full trial protocol can be accessed, if available	Supplementary File 2 Page 2
Funding	25	Sources of funding and other support (such as supply of drugs), role of funders	Supplementary File 2 Page 13

*We strongly recommend reading this statement in conjunction with the CONSORT 2010 Explanation and Elaboration for important clarifications on all the items. If relevant, we also recommend reading CONSORT extensions for cluster randomised trials, non-inferiority and equivalence trials, non-pharmacological treatments, herbal interventions, and pragmatic trials. Additional extensions are forthcoming: for those and for up to date references relevant to this checklist, see www.consort-statement.org.

TRIALS

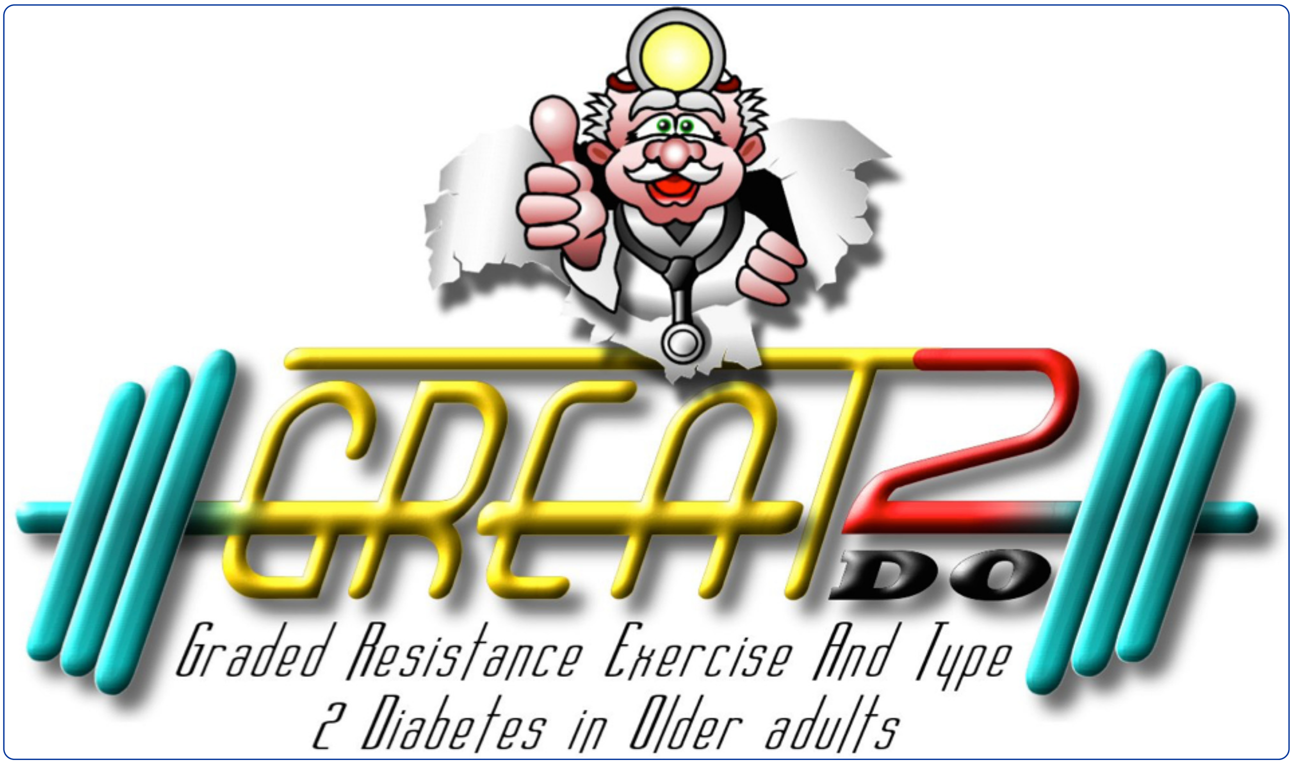

Graded Resistance Exercise And Type 2 Diabetes in Older adults (The GREAT2DO study): methods and baseline cohort characteristics of a randomized controlled trial

Simpson *et al.*

BioMed Central

Simpson *et al. Trials* (2015) 16:512
DOI 10.1186/s13063-015-1037-y

Simpson et al. Trials (2015) 16:512
DOI 10.1186/s13063-015-1037-y

TRIALS

METHODOLOGY Open Access

Graded Resistance Exercise And Type 2 Diabetes in Older adults (The GREAT2DO study): methods and baseline cohort characteristics of a randomized controlled trial

Kylie A. Simpson[1*], Yorgi Mavros[1], Shelley Kay[1], Jacinda Meiklejohn[1], Nathan de Vos[2], Yi Wang[3], Qianyu Guo[1], Renru Zhao[1], Mike Climstein[1], Bernard T. Baune[4], Steven Blair[5], Anthony J. O'Sullivan[6], David Simar[7], Nalin Singh[1] and Maria A. Fiatarone Singh[1,8,9]

Abstract

Background: Type 2 diabetes (T2D) is projected to affect 439 million people by 2030. Medical management focuses on controlling blood glucose levels pharmacologically in a disease that is closely related to lifestyle factors such as diet and inactivity. Physical activity guidelines include aerobic exercise at intensities or volumes potentially unreachable for older adults limited by many co-morbidities. We aim to show for the first time the efficacy of a novel exercise modality, power training (high-velocity, high-intensity progressive resistance training or PRT), in older adults with T2D as a means for improving glycemic control and targeting many associated metabolic and physiological outcomes.

Eligibility criteria included community-dwelling men and women previously diagnosed with T2D who met the current definition of metabolic syndrome according to the International Diabetes Federation. Participants were randomized to a fully supervised power training intervention or sham exercise control group for 12 months. Intervention group participants performed whole body machine-based power training at 80%1RM, 3 days per week. The control group undertook the same volume of non-progressive, low-intensity training. Participants were assessed at baseline, 6 months and 12 months and followed for a further 5 years, during which time participants were advised to exercise at moderate-high intensity. Glycemic control (HbA1c) and insulin resistance as measured by the homeostatic model assessment 2 (HOMA2-IR) were the primary outcomes of the trial. Outcome assessors were blinded to group assignment and participants were blinded to the investigators' hypothesis regarding the most effective intervention.

Results: We recruited 103 participants (48.5 % women, 71.6 ± 5.6 years). Participants had 5.1 ± 1.8 chronic diseases, had been diagnosed with T2D for 8 ± 6 years and had a body mass index (BMI) of 31.6 ± 4.0 kg/m^2. Fasting glucose and insulin were 7.3 ± 2.4 mmol/L and 10.6 ± 6.3 mU/L, respectively. HbA1c was 54 ± 12 mmol/mol. Eighty-six participants completed the 12-month assessment and follow-up is ongoing. This cohort had a lower-than-expected dropout (*n* = 14, 14 %) over the 12-month intervention period.

(Continued on next page)

* Correspondence: kylie.simpson@sydney.edu.au
[1]Faculty of Health Science, Exercise, Health and Performance Faculty Research Group, University of Sydney, 75 East St, Lidcombe, NSW 2750, Australia
Full list of author information is available at the end of the article

BioMed Central

© 2015 Simpson et al. **Open Access** This article is distributed under the terms of the Creative Commons Attribution 4.0 International License (http://creativecommons.org/licenses/by/4.0/), which permits unrestricted use, distribution, and reproduction in any medium, provided you give appropriate credit to the original author(s) and the source, provide a link to the Creative Commons license, and indicate if changes were made. The Creative Commons Public Domain Dedication waiver (http://creativecommons.org/publicdomain/zero/1.0/) applies to the data made available in this article, unless otherwise stated.

(Continued from previous page)

Conclusions: Power training may be a feasible adjunctive therapy for improving glycemic control for the growing epidemic of T2D in older adults.

Trial registration: Australian and New Zealand Clinical Trials Registry ACTRN12606000436572 (24 September 2006).

Keywords: Type 2 diabetes, Resistance training, Weight lifting, Randomized controlled trial, Power training

Background

Physical activity is a critical component of optimal management for the increasing epidemic of type 2 diabetes (T2D) [1]. Although not as well studied as aerobic exercise in this cohort [2], progressive resistance training (PRT) has been shown to improve glucose homeostasis in T2D, with a clinically relevant effect size (ES) not significantly different from aerobic exercise or combined exercise or lifestyle programs [3]. It may also improve blood pressure, dyslipidemia, markers of inflammation and catabolism, and visceral obesity, thus addressing many components of metabolic syndrome [4–6]. Additionally, PRT has beneficial effects on functional exercise capacity [7, 8], osteoarthritis [9], bone health [10], depression and insomnia [11], and cognitive impairment [12], thus addressing many common co-morbidities in older adults with T2D and obesity. Importantly, PRT, in contrast to aerobic exercise, attenuates or prevents the loss of lean tissue accompanying dietary weight loss [13], thus addressing the potential adverse metabolic and clinical effects such a loss of muscle and bone mass may produce [14].

Resistance training is commonly recommended at moderate intensity and slow velocity for older adults [15]. By contrast, power training typically involves pushing a weight as quickly as possible during the concentric phase and slowly returning the weight to the start position during the eccentric phase [16]. As the rate of decline in power is greater than the rate of decline in strength with aging [17], power training may be particularly important to older adults, and has notably been shown to be more effective in improving physical function [18] and maintaining bone density [19] as compared to conventional slow-velocity resistance training. Power training has also been shown to improve functional tasks such as standing up from a chair, climbing stairs [20] and gait speed [21]. Moreover, power training specifically targets the type 2b muscle fiber atrophy that predominates in older adults and contributes to sarcopenia [22–24]. Power training thus has the potential to provide a broader spectrum of benefit than slow-velocity PRT, yet this alternative form of PRT has never been directly tested as a strategy to treat individuals with diabetes and metabolic syndrome.

With any new therapy, it is important to establish the overall risk/benefit ratio, rather than just focus upon the effect on the target symptom. Thus, our measures of other clinical problems in these individuals with metabolic syndrome, such as muscle weakness, immobility, disability, depression, cognitive impairment, sleep disturbance, postural and post-prandial hypotension, and cardiovascular symptoms will provide a balanced perspective on the unique benefits and risks of this therapeutic approach compared to usual care. It is also critically important to establish the utility of this intervention on its own, before combining it with other interventions such as weight loss diets, or aerobic exercise [4, 25] so that the independent contribution of power training to diabetes and metabolic syndrome can be definitively established.

Objectives

Our specific aim was to conduct a 12-month randomized controlled trial (RCT) to test the efficacy of power training added to the usual medical care of older adults with T2D and metabolic syndrome. In addition, the long-term feasibility and benefits of this type of exercise will be assessed over 6 years of follow-up.

Primary hypothesis

Power training will be associated with sustained improvements in insulin sensitivity and HbA1c compared to the sham exercise control group at 6-month and 12-month follow-up.

Secondary hypotheses

1. Power training will be associated with improvements in the other components of metabolic syndrome and cardiovascular risk associated with T2D, including: increased circulating high-density lipoprotein (HDL) cholesterol, decreased total and low-density lipoprotein (LDL) cholesterol and triglyceride (TG) levels, decreased ambulatory blood pressure, increased aerobic capacity, improved heart rate variability and postural hypotension compared to the sham exercise control condition.
2. Power training will be associated with a significant reduction in visceral adiposity and intramuscular lipid and increase in regional and whole body measures of lean muscle mass, bone mineral density, function, and metabolism, as well as a shift in the

anabolic milieu (decreased circulating and adipose tissue levels of inflammatory/catabolic cytokines and increased anabolic/anti-inflammatory factors) compared to the sham exercise control condition.
3. Body composition changes across the entire cohort will be related to observed metabolic/inflammatory improvements, whereas total body mass changes will not.
4. Randomization to the power training intervention as well as higher levels of participation in structured physical activity during follow-up will predict better outcomes in the above primary and secondary domains across the 6-year follow-up.

Methods

Recruitment

Sample size estimates

Hypothesized differences between the experimental and control participants for the primary outcomes drove sample size estimates of insulin resistance and HbA1c, based on published studies of PRT in diabetes/obesity [6, 26]. Largest available standard deviations were used for conservative estimates of ES. The most comprehensive meta-analysis at the time of study planning [27] indicated that control participants in enrolled exercise trials did not improve in metabolic outcomes, thus the control change was set at 0. Setting beta at 0.2, and alpha at 0.05, and n1 = n2, the following sample size requirements were estimated for changes from baseline at 12 months (Table 1).

Thus, 98 participants recruited, (assuming 20 % attrition), would provide 78 participants at 12 months, as required by the smaller ES hypothesized for HbA1c. Based on our pilot work, we estimated we would need to screen approximately 500 participants by phone, and 125 in person, to enroll approximately 100 eligible/interested participants. We anticipated that our loss to follow-up would actually be less than 20 %.

Recruitment strategies

Participants were recruited via general practitioner referral, targeted mail-outs, advertisements in local newspapers and seniors' magazines and from brochures distributed to local medical practitioners and pharmacies. Participants were recruited from August 2006 to December 2010 with the final 12-month assessment in December 2011. Follow-up testing is due for completion in February 2016.

Management

All incoming responses were recorded by the research assistant and logged in a tracking database that outlined the flow of participants through the recruitment, screening and (if eligible) intervention process.

Screening

Potential participants contacted the research assistant and a telephone interview was conducted or scheduled for a convenient time. The telephone screening was comprised of questions to ascertain the participant's basic demographic and contact information, current diabetic and health status, medical history, current medication use and physical activity/exercise levels. The study physician reviewed all of the individual screenings and participants were subsequently notified of their eligibility. If eligible, participants were requested to attend the University of Sydney for subsequent testing (including a health and physical examination by a physician and a 12-lead electrocardiogram (ECG) and maximal exercise stress test) to determine their eligibility.

Participants

Eligibility criteria for participants

The inclusion and exclusion criteria are listed in Fig. 1. Participants eligible for inclusion were treated with diet alone, oral medications or insulin or combination at the time of enrollment. Metabolic syndrome was defined according to the International Diabetes Federation as:

- Central obesity (defined as waist circumference ≥ 94 cm for Europid men and ≥ 80 cm for Europid women, plus any 2 of the following 4 factors:
- Raised TG level: ≥ 1.7 mmol/L, or specific treatment for this lipid abnormality
- Reduced HDL cholesterol: < 1.03 mmol/L) in men or < 1.29 mmol/L in women, or specific treatment for this lipid abnormality
- Raised resting blood pressure: systolic blood pressure (BP) ≥ 130 mmHg or diastolic BP ≥

Table 1 Primary outcomes and effect sizes

Primary outcome	Experimental mean change	Sham control Mean change	Pooled standard deviation	Effect size (*d*)	Sample required (total cohort)
HOMA2-IR	−0.6	0	0.8	0.75	52
HbA1c (mmol/mol)	−13.1	0	18.6	0.70	78

Sample size estimates were driven by hypothesized differences between the experimental and control participants in the primary outcomes of the trial: insulin resistance and glucose homeostasis, as measured using HOMA2-IR and HbA1c respectively. Effect sizes were calculated based on an average of published studies of PRT in diabetes/obesity [6, 26].
HbA1c glycosylated hemoglobin, *HOMA2-IR* homeostatic model of assessment of insulin resistance 2

Inclusion Criteria	Exclusion Criteria
▪ Age >60 years<br>▪ Previously diagnosed T2D<br>▪ Clinically stable T2D (no change in medications in the previous 2 months)<br>▪ BMI >25 kg/m^2<br>▪ Abnormal lipid profile<br>▪ Sedentary (no PRT; structured exercise ≤ 1/week; low-moderate intensity walking or other aerobic exercise ≤ 150 min/week)	▪ Significant cognitive impairment<br>▪ Non-ambulatory<br>▪ Lower extremity amputation (other than toes)<br>▪ Alcohol or substance abuse<br>▪ Inability to comply with study requirements over the course of one year<br>▪ Unstable cardiovascular disease<br>▪ Unrepaired aortic aneurysm<br>▪ Proliferative diabetic retinopathy<br>▪ Rapidly progressive terminal illness
T2D = Type 2 Diabetes; BMI = Body Mass Index; PRT = Progressive Resistance Training.	

Fig. 1 Inclusion and exclusion criteria. *BMI* body mass index, *PRT* progressive resistance training, *T2D* type 2 diabetes

85 mmHg, or treatment of previously diagnosed hypertension

- Raised fasting plasma glucose (FPG) ≥ 5.6 mmol/L, or previously diagnosed T2D.

Exclusion criteria included significant cognitive impairment (inability to comprehend informed consent or evidence of functional impairment related to cognition on screening), non-ambulatory status or lower extremity amputation other than toes, current alcohol or substance abuse, inability to comply with study requirements over the course of 1 year due to travel plans or other commitments, or specific contraindications to resistance training exercise, such as unstable cardiovascular disease, aortic aneurysm, proliferative diabetic retinopathy, uncontrolled hypertension, or rapidly progressive or

terminal illness. Temporary exclusions included recent laser or other ocular surgery (within 2 weeks), symptomatic hernias, acute illness, or recent fracture or other injury, until resolved.

Settings and location where data were collected

All assessments were carried out at the University of Sydney, Faculty of Health Sciences, Lidcombe and in the Radiology Department of Royal Prince Alfred Hospital, Sydney. Training sessions were conducted at the Harbord Diggers' Freshwater Fitness Center, Harbord and at The Center for STRONG Medicine, Balmain Hospital, Balmain.

Interventions

Experimental intervention: power training

Experimental participants performed power training 3 days per week and using 8 major muscle groups on pneumatic resistance under supervision. We used a version of PRT called power training, in which the concentric contraction (lifting the weight) was done as fast as possible, and the lowering of the weight was done slowly (over 2–3 seconds). The exercises targeted the majority of the large muscle groups of the arms, legs, and trunk and consisted of lateral pulldown, chest press, upper back, leg press, knee extension, and knee flexion, hip extension and hip abduction. These are symmetrical muscle groups and functionally relevant to the activities of daily living, gait, and balance of older adults. For each exercise, participants performed 3 sets of 8 repetitions with a fast concentric and slow eccentric phase on pneumatic resistance training machines (approximately 6 seconds per repetition with 2 minutes of rest between sets), a regimen which has been shown to produce optimum adaptations in terms of muscle power, strength, endurance in older adults [16].

The intensity was set at 80 % of the most recently determined peak strength (1 repetition maximum or 1RM). Resistances used were increased as tolerated using the Borg Scale Rating of Perceived Exertion [28, 29] on a continuous basis throughout the 12 months, and 1RM testing was repeated at 2-week intervals to ascertain progress and regulate intensity. All training was fully supervised by skilled exercise physiologists to maintain proper intensity and progression, as 2 separate trials in T2D have now shown that metabolic benefits of PRT disappear when participants are switched to semi-supervised community-based or home-based training [30, 31]. This was attributed to a drop in adherence and intensity when supervision was withdrawn. As this is a first-time efficacy trial of power training, our intent was to maximise protocol adherence.

Control group intervention: sham PRT

The same trainers supervised sham exercise control participants in the same facility but at different hours to avoid contamination and unblinding. These participants performed three sets of eight repetitions on the same machines, with no loading beyond the weight of the bar of the machine, using slow concentric and eccentric contraction speed. No interim 1RM testing and no progression took place. We have found that similar regimens do not improve muscle function or mass, functional status, mobility, depression, aerobic capacity, or other clinical outcomes we are measuring [32, 33]. Low-intensity resistance training has also been shown to have no effect on visceral fat [34], adiponectin [35], glucose homeostasis or insulin sensitivity, or bone mineral density [36], thus providing an ideal sham exercise control condition.

Outcomes

The primary outcomes of this study were insulin resistance 96 hours after the last exercise bout as assessed by the homeostatic model assessment 2 (HOMA2) computer model and HbA1c. The 96-hour time interval for HOMA2 was chosen to minimize the well-described acute bout effect of exercise on insulin sensitivity, as we are primarily interested in long-term training adaptations related to visceral fat/muscle mass.

Secondary outcomes and covariates include all of the components of metabolic syndrome, body composition, adipokines, muscle morphology and metabolism, genetic and epigenetic markers related to metabolic/cardiovascular health and exercise adaptation, measures of energy expenditure and fat oxidation, neuropsychological function, cardiovascular health status, quality of life, dietary intake and habitual physical and sedentary activity levels. Blinded assessors, at a laboratory facility separate from the training site to prevent unblinding, repeated all measurements at baseline, 6 months, and 12 months in experimental and control participants. In addition, selected measures were repeated annually during the 5 additional years of follow-up.

Domains of assessment

All outcome measures administered by blinded assessors at baseline, 6-month and 12-month follow-up are listed in Table 2. During the intervention phase, assessments were conducted over 3 different days to allow for the correct timing of exercise bouts and blood draws and to ensure adequate participant preparation (e.g. the cessation of blood thinning medication where possible). Figure 2 details the timing of assessments throughout the course of the study.

Table 2 Outcome measures

Outcome	Outcome measure	Description
Primary	Insulin sensitivity	72 hours and 96 hours post exercise
		• HOMA2 computer model for insulin sensitivity (IR, %S) [53]
		• HOMA2 computer model for beta cell function (%Beta)
	Glucose homeostasis	Fasting glucose
		Glycated hemoglobin [53]
		Insulin
		C-peptide levels
		Diabetic medication inventory and dosages
		Meal tolerance test
Secondary	Cardiovascular health	Resting heart rate variability and pulse wave velocity (arterial stiffness)
		24-hour ambulatory blood pressure
		Postural blood pressure
		Ankle brachial blood pressure index
	Lipid metabolism	Total cholesterol
		Low/High-density cholesterol
		Triglycerides
		Basal fat oxidation via indirect calorimetry
		Intramuscular lipid content
	Muscle morphology and metabolism	Muscle tissue biopsy (vastus lateralis) [54]
		• Glucose transporter type 4 receptor protein
		• Intramuscular insulin-like growth factor 1
		• Glycogen content
		• Muscle fiber cross-sectional area
		• Muscle fibrertyping
	Adipokines and Inflammatory markers	Adipose tissue biopsy [54]
		• TNF-α
		• Interleukin-6
		• High molecular weight adiponectin
		• C-Jun N-terminal kinase
		• Serum C-reactive protein [55]
	Body composition [53]	Bioelectrical impedance analysis
		• Skeletal muscle mass, skeletal muscle mass index
		• Fat free mass
		• Fat mass
		• Body fat percentage
		Computed tomography scan
		• Abdominal and thigh girth, sagittal diameter
		• Abdominal total, visceral and subcutaneous fat area
		• Thigh total, intramuscular and subcutaneous fat area and muscle density (intramuscular lipid index)
		• Thigh muscle area
		Waist circumference
		Body mass index

Table 2 Outcome measures *(Continued)*

	Exercise capacity and Functional status	Exercise stress test (maximal treadmill test with indirect calorimetry)
		• Peak aerobic capacity
		• Anaerobic threshold
		• Oxygen uptake efficiency slope
		• Heart rate recovery
		Muscle strength, power and endurance
		Habitual and maximal gait speed
		Static and dynamic balance
		Chair stand and stair climb power
	Neuropsychological profile	Geriatric Depression Scale
		Pittsburgh Sleep Quality Index
		Ewart's Self-efficacy Scale
		Cognitive function
		• Mini Mental State Exam
		• Word List Recognition and Recall
		• Trail Making A and B
		Actigraph sleep architecture over 7 days
		• Total time in/out of bed
		• Sleep latency, sleep efficiency
		• Total time asleep
		• Number of awakenings
	Health-related quality of life	Medical Outcomes Study 36-Item Short-Form Health Survey
	Nutritional intake	Food frequency questionnaire of Bloch over past 4 months
	Energy expenditure	Resting metabolic rate via indirect calorimetry
		Habitual physical activity and sedentary behaviour via Physical Activity Scale for the Elderly
		Actigraph accelerometers over 7 days
		• Waking hours
		• Wear time
		• Total sedentary time, mean sedentary time, median sedentary time
		• Percentage of time spent in sedentary (1 MET), light (<3 MET), moderate (3–6 MET) and vigorous (>6 MET) activity
		• Total energy expenditure spent in sedentary (1 MET), light (<3 MET), moderate (3–6 MET) and vigorous (>6 MET) activity
		• Physical activity level (PAL score)

%S percent sensitivity, *HOMA2* homeostatic model assessment 2, *MET* metabolic equivalent of task, *TNF-α* tissue necrosis factor alpha

Randomization

Sequence generation

A computerized random-number generator (http://www.randomization.com, created by Dr Gerard E. Dallal, Tufts University) was used to randomize eligible participants at the level of the individual participant, stratified by sex and use of insulin, in blocks of four.

Allocation concealment

Sealed envelopes were prepared by an independent researcher, containing sequential treatment assignments based on a computer-generated randomization scheme, and opened by the participant's trainer after completion of all baseline testing.

Implementation

The study was approved by the University of Sydney and the Sydney South West Area Health Service Human Research Ethics Committees (RPA HREC protocol number × 04-009602/08/2006). A blinded outcomes assessor obtained informed consent from all participants enrolled in the study and informed the independent researcher

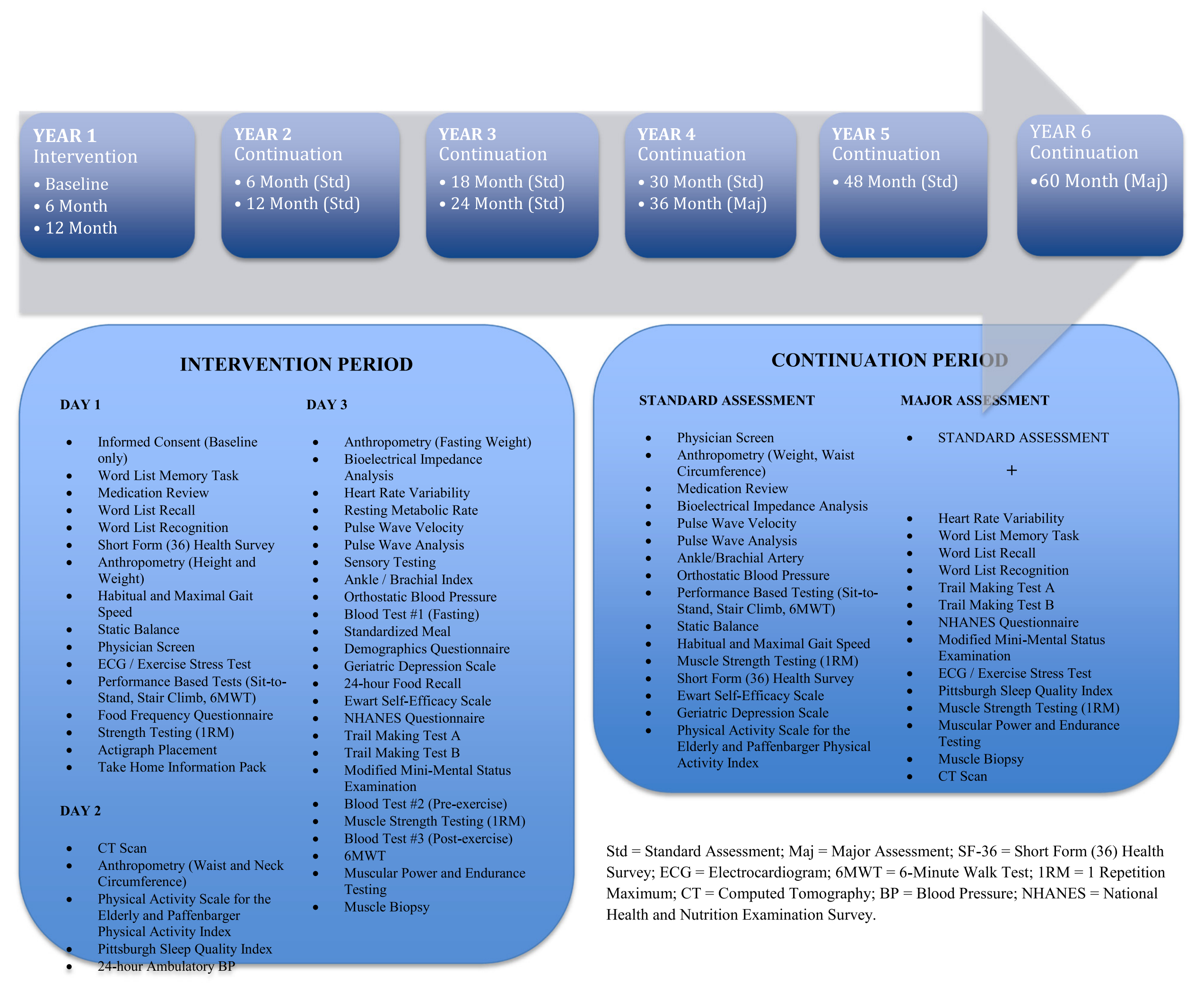

Fig. 2 Timeline of assessments. **a** 6-month and 12-month assessments performed 72 hours after previous training session. **b** 6-month and 12-month assessments performed 48 hours after previous training session. **c** 6-month and 12-month assessments performed 96 hours after previous training session. *1RM* 1 repetition maximum, *6MWT* 6-Minute Walk Test, *BP* blood pressure, *CT* computed tomography, *ECG* electrocardiogram, *SF-36* 36-Item Short-Form Health Survey, *NHANES* National Health and Nutrition Examination Survey

when the participant was eligible for randomization (after completion of baseline testing). Participants were notified of their training session time by the exercise physiologist administering the intervention.

Blinding

This is the first truly double-blind sham exercise-controlled RCT in T2D. All outcomes assessors were blinded to treatment assignment for the duration of the study. Only the exercise physiologists and medical practitioners responsible for administering and overseeing the exercise programs were informed of participant group assignment. Participants were also blinded to the investigators' hypothesis and only informed of their training time, not group assignment as active or sham, and were told that two different kinds of exercise were being compared for efficacy.

Statistical methods

All outcomes will be analyzed using all available data without imputation for missing time points via repeated measures linear mixed models, without regard to discontinuation or dropout. Secondary per-protocol analyses will be carried out on participants with ≥ 70 % compliance to exercise. All mixed models will be adjusted for relevant covariates if necessary, with group by time interaction as the primary outcome of interest. Covariates will be chosen as appropriate via a priori

hypothesized confounders if there are differences between groups within relevant variables at baseline within the cohort despite randomization. Relationships between variables of interest at baseline between changes scores will be determined by simple and stepwise regression models and logistic regression models as appropriate. Statistical significance will be initially assumed at the 0.05 level, as all hypotheses were specified a priori, and Bonferroni correction is considered overly conservative in this instance [37]. Effect sizes and 95 % confidence intervals (CIs) will be calculated for all outcomes. Calculations of ES will be adjusted via Hedges' bias-corrected ES for small sample sizes [38] and interpreted according to Cohen's interpretation of "trivial" (<0.20), "small" (≥0.20 < 0.50), "moderate" (≥0.50 < 0.80), and "large" (≥0.80) ES [39]. Ninety-five percent CIs for the relative ES will calculated. For ES in non-normally distributed data, median will substituted for mean, and range/4 will be substituted for SD.

Results

Participant flow

Participants were assessed at baseline, 6 and 12 months for the intervention phase of study and annually for the remaining 5-years of follow-up. Figure 3 shows the flow of participants through the study.

Recruitment

We assessed 427 people for eligibility (Fig. 3). After completing the telephone screening questionnaire, 324 people were deemed ineligible for not meeting the inclusion criteria ($n = 285$), declining to participate ($n = 15$), and a further 24 people were either unable to commit, had transport difficulties/did not live local to the training sites or had medical contraindications. A total of 103 people gave consent, met the eligibility criteria and were randomized to either the power training intervention group ($n = 49$) or the sham-exercise control group ($n = 54$).

Losses and exclusions

Three participants withdrew from the study prior to commencing the intervention. Fourteen participants dropped out of the study (did not complete follow-up assessments) after commencing the intervention (intervention group $n = 10$; control group $n = 4$). One was an adverse event related to the intervention (musculoskeletal injury in the intervention group), five felt the intervention was too hard, four were medical concerns unrelated to the intervention, three were due to commitment issues, and one was due to disinterest. Our attrition of 14 % at 12 months was less than expected for the intervention period. As expected, there has been a steady decline in participation over the follow-up phase. A total of 70 people commenced the 5-year post-intervention program with a total of 41 participants expected to complete the entire 6 years of follow-up. Currently, 35 participants have completed the 6-year follow-up, with the remaining 6 participants scheduled for their final assessment.

Baseline demographics

The baseline participant characteristics are reported in Table 3. Forty-nine and 54 participants were randomized to the invervention and control groups, respectively. Our eligibility criteria were designed so as to maximize external validity as only terminal or unstable diseases, or conditions which would permanently preclude resistance training, were exclusionary. Thus, our cohort was generally representative of older diabetic cohorts in Australia and other countries, with multiple co-morbidities and chronic medication usage. For example, the Australian Institute for Health and Welfare 2007–8 [40] report states that 58 % of individuals with diabetes also have cardiovascular disease. In our cohort, nearly one half (49 %) were being treated for cardiovascular disease. Similarly, 43 % of the Graded Resistance Exercise And Type 2 Diabetes in Older adults (GREAT2DO) cohort had hypercholesterolemia at baseline, which is not dissimilar to the Australian adult population where half of all adults over 25 years of age have high total cholesterol levels (data from 1999–2000) [41]. Among the 103 participants in this study, only 5.8 % were current smokers, similar to the 4.4 % reported in the Look AHEAD study of adults with T2D in 2006 [42]. Sixteen participants reported insulin use for control of their diabetes. In comparison to some previous trials that have excluded people with cardiovascular disease and insulin therapy, overall our cohort is more representative of the general population of older adults with T2D.

Adverse events

There were eight adverse events in six participants adjudicated by the study physician to be related to study procedures; seven in the intervention group and one in the control group. These included three syncopal episodes in a male participant with known syncope, one hamstring strain, one back pain leading to dropout, one exacerbation of pre-existing umbilical hernia requiring surgical repair, one subscapularis tear in a female participant with pre-existing grade IV osteoarthritis/rotator cuff disease requiring surgery, and one partial thickness tear of a rotator cuff muscle managed conservatively.

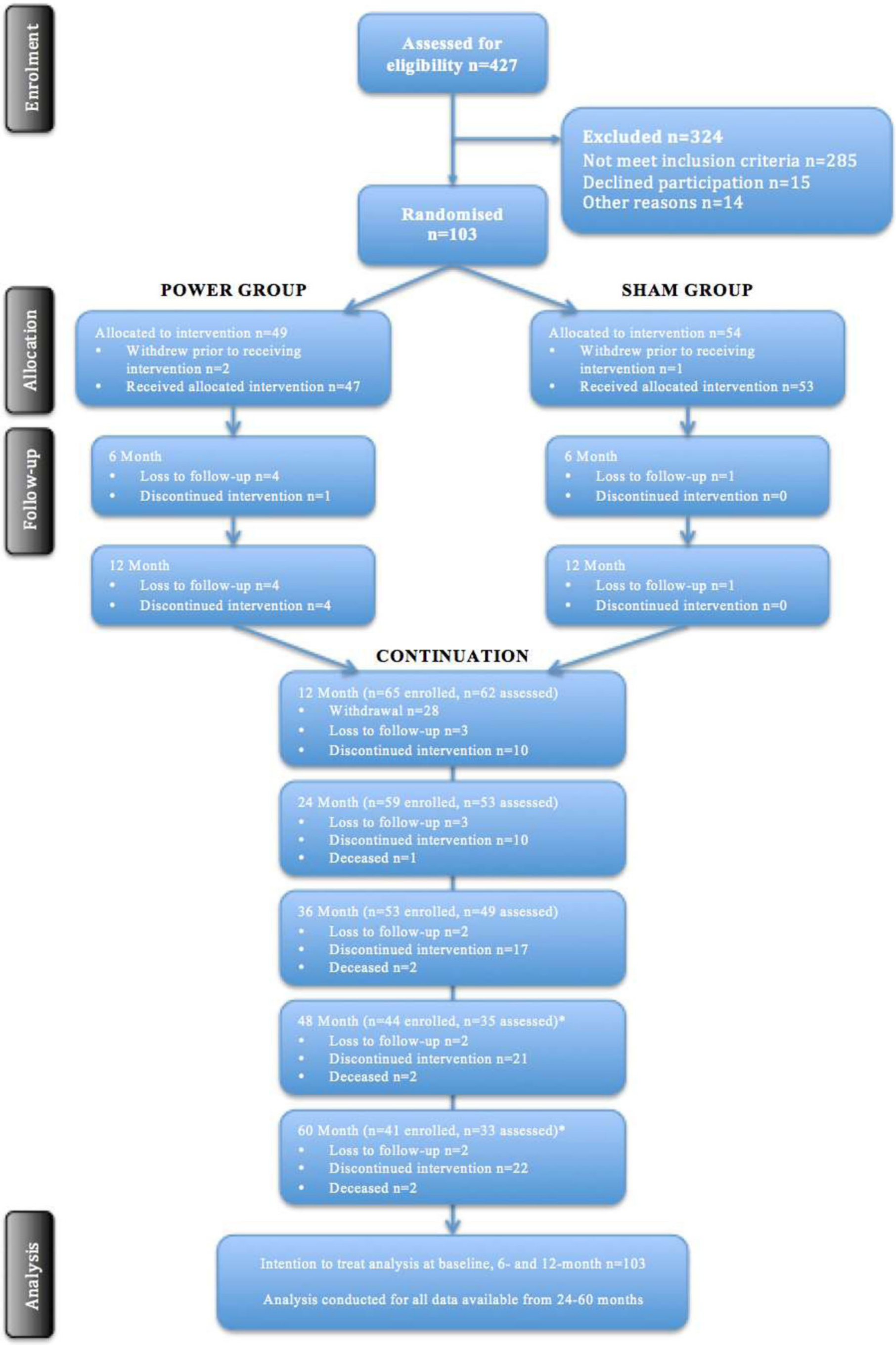

Fig. 3 Consolidated Standards of Reporting Trials (CONSORT) flow chart, Loss to follow-up and deceased numbers are cumulative. The numbers for missed assessments are specific to each time point. A linear mixed-effects model with repeated measures will be used to determine changes over time as this method allows for all available data to be used without imputation for missing values. Thus, even with dropouts, all participants who entered the study at baseline will be entered into the model

Discussion

We have shown for the first time that it is feasible to recruit and retain older adults with T2D and multiple chronic co-morbidities in a long-term trial of high-intensity power training. Few RCTs of PRT, as an isolated addition to usual care in middle-aged and older adults with T2D, have been published: however, the magnitude of change in glucose control (HbA1c) in these studies was comparable to the effect of aerobic exercise or oral hypoglycemic therapy [3]. One study,

Table 3 Baseline participant characteristics

	Power	Sham	Total
	(n = 49)	(n = 54)	(n = 103)
Demographics			
Age (years)	66.9 ± 4.7	68.8 ± 6.1	67.9 ± 5.5
Sex (female)	24.0 (49.0)	27.0 ± (50.0)	51.0 ± 0.5
Smoking status			
Current	3.0 (6.1)	3.0 (5.6)	6.0 (5.8)
Past	31.0 (63.3)	33.0 (61.1)	64.0 (62.1)
Ethnic origin			
Caucasian	49.0 (100.0)	50.0 (92.6)	99.0 (96.1)
Asian	0.0 (0.0)	2.0 (3.7)	2.0 (1.9)
Indian	0.0 (0.0)	2.0 (3.7)	2.0 (1.9)
Chronic diseases			
Duration of diabetes (years)	6.9 ± 5.1	9.1 ± 6.7	8.0 ± 6.0
Number of chronic diseases	5.1 ± 1.9	5.1 ± 1.8	5.1 ± 1.8
Chronic diseases			
Hypertension	37.0 (75.5)	39.0 (72.2)	76.0 (73.8)
Osteoarthritis	35.0 (71.4)	33.0 (61.1)	68.0 (66.0)
Cardiovascular diseases	23.0 (46.9)	27.0 (50.0)	50.0 (48.5)
High cholesterol	24.0 (49.0)	21.0 (38.9)	45.0 (43.7)
Cancer	14.0 (28.6)	17.0 (31.5)	31.0 (30.1)
GERD (Reflux/Barrett's esophagus)	8.0 (16.3)	14.0 (25.9)	22.0 (21.4)
PVD	10.0 (20.4)	11.0 (20.4)	21.0 (20.4)
IHD, MI, Angina	8.0 (16.3)	11.0 (20.4)	19.0 (18.4)
Sleep apnea	7.0 (14.3)	12.0 (22.2)	19.0 (18.4)
Depression	10.0 (20.4)	8.0 (14.8)	18.0 (17.5)
Hypothyroidism	7.0 (14.3)	9.0 (16.7)	16.0 (15.5)
Arrhythmia	8.0 (16.3)	6.0 (11.1)	14.0 (13.6)
Ulcers (gastrointestinal tract)	7.0 (14.3)	7.0 (13.0)	14.0 (13.6)
Osteoporosis/Osteopenia	5.0 (10.2)	8.0 (14.8)	13.0 (12.6)
Gout	7.0 (14.3)	5.0 (9.3)	12.0 (11.7)
Benign prostatic hyperplasia	3.0 (6.1)	8.0 (14.8)	11.0 (10.7)
Chronic venous disease	4.0 (8.2)	6.0 (11.1)	10.0 (9.7)
COPD/CAL	2.0 (4.1)	7.0 (13.0)	9.0 (8.7)
Hyperthyroidism	2.0 (4.1)	6.0 (11.1)	8.0 (7.8)
Esophagitis	5.0 (10.2)	3.0 (5.6)	8.0 (7.8)
Health status			
Weight (kg)	89.5 ± 15.2	88.8 ± 18.8	89.1 ± 17.1
BMI (kg/m^2)	31.5 ± 4.7	31.6 ± 6.0	31.6 ± 5.4
Waist circumference (cm)			
Men	112.4 ± 9.6	109.3 ± 11.5	110.8 ± 11.7
Women	109.6 ± 9.6	108.7 ± 11.5	109.1 ± 12.6
Resting blood pressure (mmHg)			
Systolic	147.2 ± 17.9	145.1 ± 17.9	146.1 ± 17.9
Diastolic	78.9 ± 7.5	77.4 ± 10.2	78.1 ± 9.0

Table 3 Baseline participant characteristics *(Continued)*

Resting heart rate (bpm)	65.2 ± 12.1	64.6 ± 8.4	64.9 ± 10.2
Fasting glucose (mmol/L)	7.4 ± 2.5	7.1 ± 2.2	7.3 ± 2.4
Fasting insulin (mU/L)	10.1 ± 5.9	11.1 ± 6.7	10.6 ± 6.3
HbA1c (%)	6.9 ± 0.9	7.3 ± 1.3	7.1 ± 1.1
HOMA2-IR	2.6 ± 1.0	2.9 ± 1.3	2.7 ± 1.1
Cholesterol (mmol/L)			
Total	4.5 ± 1.1	4.2 ± 1.1	4.4 ± 1.1
HDL	1.2 ± 0.3	1.2 ± 0.4	1.2 ± 0.3
LDL	2.5 ± 0.9	2.3 ± 0.9	2.4 ± 0.9
Triglycerides	1.7 ± 1.0	1.7 ± 0.9	1.7 ± 0.9
C-reactive protein (mg/L)	3.8 ± 3.2	4.5 ± 4.9	4.2 ± 4.2
Diabetic treatment			
Diet only	8.0 (16.0)	10.0 (19.0)	18.0 (17.0)
Oral hypoglycemics only	34.0 (69.0)	35.0 (65.0)	69.0 (67.0)
Oral hypoglycemics and Insulin	4.0 (8.0)	7.0 (13.0)	11.0 (11.0)
Insulin only	3.0 (6.0)	2.0 (4.0)	5.0 (5.0)
Physical Function			
Habitual gait speed (m/s)	1.2 ± 0.2	1.2 ± 0.2	1.2 ± 0.2
Maximal gait speed (m/s)	1.9 ± 0.3	1.9 ± 0.3	1.9 ± 0.3
6MWT (m)	551.1 ± 93.8	539.1 ± 95.3	544.8 ± 94.3

Values are means ± SD or *n* (%)
6MWT 6-Minute Walk Test, *BMI* body mass index, *BPH* benign prostatic hyperplasia, *COPD/CAL* chronic obstructive pulmonary disease/chronic airflow limitation, *GERD* gastro-esophageal reflux disease, *HbA1c* glycemic control, *HDL* high-density lipoprotein, *HOMA2-IR* homeostatic model assessment 2, *IHD* ischemic heart disease, *LDL* low-density lipoprotein, *MI* myocardial infarction, *PVD* peripheral vascular disease,

which directly compared isolated PRT to aerobic exercise in middle-aged type 2 adults with diabetes [26], found that PRT significantly improved 48-hour continuous glucose control, HbA1c, insulin sensitivity, and lipids, whereas aerobic exercise was ineffective. Limitations of previous studies of PRT include small sample sizes and relatively short periods of follow-up in some cases, and lack of comprehensive assessment of mechanisms of benefit, cardiovascular profile and associated clinical benefits relevant to older adults.

Thus, despite the strong theoretical rationale for its use in this cohort, and its recent advocacy by expert consensus panels internationally [1, 14, 43], PRT is not a common treatment for diabetes [2] and more evidence of feasibility, safety, and efficacy are needed. This need is highlighted by the results of the Look AHEAD trial, which reported no difference in the primary outcome of cardiovascular events following lifestyle modification in type 2 diabetics, despite clear benefits in secondary outcomes such as weight, physical activity level, fitness, quality of life, depression levels and metabolic risk [44–46]. However, most lifestyle interventions, even intensive ones such as Look AHEAD, may not attenuate losses of lean tissue mass seen with both aging and caloric restriction. In fact, bone losses were greater in men in the intensive lifestyle group in Look AHEAD compared to controls, and were proportional to weight loss achieved [47, 48]. This important issue of lean tissue loss accompanying lifestyle modification programs that do not prioritize or include anabolic exercises thus requires additional study, and is the focus of the GREAT2DO trial described here.

Conclusion

To our knowledge no study investigating high-intensity power training has yet been published, and thus the GREAT2DO study will provide the first evidence of the safety, efficacy and long-term feasibility of this novel modality of anabolic exercise in older adults with T2D. Other studies suggest that power training is useful for osteoporosis [49, 50], balance [51], functional performance [18] and quality of life [52]. If our hypotheses are correct, improvement in metabolic health and other secondary outcomes from this trial may add to the growing rationale for this unique and robust form of exercise training for the treatment of chronic disease-related and age-related syndromes.

Abbreviations

1RM: 1 repetition maximum; 6MWT: 6-Minute Walk Test; BMI: body mass index; BP: blood pressure; BPH: benign prostatic hyperplasia; COPD/CAL: chronic obstructive pulmonary disease/chronic airflow limitation; CT: computed tomography; ECG: electrocardiogram; FPG: fasting plasma glucose; GERD: gastro-esophageal reflux disease; GREAT2DO: Graded Resistance Exercise And Type 2 Diabetes in Older adults; ES: effect size; HbA1c: glycemic control; HDL: high-density lipoprotein; HOMA: homeostatic model assessment; IHD: ischemic heart disease; IR: insulin resistance; LDL: low-density lipoprotein; MI: myocardial infarction; NHANES: National Health and Nutrition Examination Survey; PRT: progressive resistance training; PVD: peripheral vascular disease; RCT: randomized controlled trial; SF-36: Medical Outcomes Study 36-Item Short-Form Health Survey; T2D: type 2 diabetes; TG: triglycerides.

Competing interests

There are no known competing interests associated with publication of this manuscript, and to our knowledge no commercial interests which will benefit.

Authors' contributions

All authors read and approved the final manuscript. In addition, other specific roles are outlined below: KAS, primary blinded outcomes assessor, participant recruitment, maintained operating procedures and wrote the manuscript; SK, assisted with participant training and was responsible for CT scan data collection and analysis; YM, assisted with data collection, participant training and data analysis, reviewed and edited the manuscript; JM, assisted with participant training, database management and accelerometer analysis; NV, assisted with participant training; YW, assisted with data collection and muscle biopsy sampling and analysis; QG, assisted with data collection and muscle biopsy sampling and analysis of muscle biopsy outcomes; RZ, assisted with participant training, database management and analysis; MC, study design and expert opinion, supervision of training and edited/reviewed the manuscript; BB, study design and expert opinion, cytokine analyses; SB, study design and expert opinion, acquisition of funding; AO, study design and expert opinion, acquisition of funding, metabolic outcome analytic design; DS, study design and expert opinion, muscle biopsy analyses; NS, study design and expert opinion, supervised training/intervention delivery; MAFS, overall coordination and supervision of the trial, study design and acquisition of funding, supervised data analyses, supervised and assisted with data collection, contributed to the discussion, and reviewed and edited the manuscript.

Acknowledgments

We would like to thank our participants for their generous contributions of time and spirit. We would like to thank Harbord Diggers' Freshwater Fitness Center and The STRONG Clinic at Balmain Hospital for the use of their gym facilities and Keiser Sports Health Ltd for donations of resistance training equipment. The Graded Resistance Exercise and Type 2 Diabetes in Older adults (GREAT2DO) study was funded by project grant #512381 from the National Health and Medical Research Council (NHMRC), grants from The Australian Diabetes Society, Diabetes Australia and the Rebecca L. Cooper Foundation. Y. Mavros was supported by the Australian Postgraduate Award Scholarship. Y. Wang was supported by the University of Sydney International Postgraduate Research Scholarship.
This study fulfilled a portion of the postgraduate degree requirements for the following students: Shelley Kay, Yi Wang, Yorgi Mavros, Renru Zhao, Vivienne Quo.

Author details

[1]Faculty of Health Science, Exercise, Health and Performance Faculty Research Group, University of Sydney, 75 East St, Lidcombe, NSW 2750, Australia. [2]The Center for STRONG Medicine, Balmain Hospital, 29 Booth St, Balmain, NSW 2041, Australia. [3]San Francisco, Diabetes Center, University of California, Box 0540513 Parnassus Ave 1119, San Francisco, CA 94143-0540, USA. [4]Discipline of Psychiatry, The University of Adelaide, Level 4, Eleanor Harrold Building, Royal Adelaide Hospital, Adelaide, SA 5005, Australia. [5]Department of Exercise Science, Public Health Research Building, University of South Carolina, 921 Assembly St, Columbia, SC 29208, USA. [6]Department of Medicine, University of New South Wales, St George and Sutherland Clinical School, St George Hospital, Gray St, Kogarah, NSW 2217, Australia. [7]Faculty of Medicine, Metabolic Disorders Research Group, University of New South Wales, Sydney, NSW 2052, Australia. [8]Sydney Medical School, University of Sydney, Sydney, NSW 2000, Australia. [9]Hebrew SeniorLife and Jean Mayer USDA Human Nutrition Center on Aging, Tufts University, Boston, MA, USA.

Received: 6 March 2015 Accepted: 27 October 2015
Published online: 10 November 2015

References

1. Colberg SR, Albright AL, Blissmer BJ, Braun B, Chasan-Taber L, Fernhall B, et al. Exercise and type 2 diabetes: American College of Sports Medicine and the American Diabetes Association: joint position statement. Exercise and type 2 diabetes. Med Sci Sports Exerc. 2010;42(12):2282–303. doi:10.1249/MSS.0b013e3181eeb61c.
2. Armstrong MJ, Colberg SR, Sigal RJ. Moving beyond cardio: the value of resistance training, balance training, and other forms of exercise in the management of diabetes. Diabetes Spectr. 2015;28(1):14–23. doi:10.2337/diaspect.28.1.14.
3. Umpierre D, Ribeiro PA, Kramer CK, Leitao CB, Zucatti AT, Azevedo MJ, et al. Physical activity advice only or structured exercise training and association with HbA1c levels in type 2 diabetes: a systematic review and meta-analysis. JAMA. 2011;305(17):1790–9. doi:10.1001/jama.2011.576.
4. Dunstan DW, Daly RM, Owen N, Jolley D, De Courten M, Shaw J, et al. High-intensity resistance training improves glycemic control in older patients with type 2 diabetes. Diabetes Care. 2002;25(10):1729–36.
5. Hameed UA, Manzar D, Raza S, Shareef MY, Hussain ME. Resistance training leads to clinically meaningful improvements in control of glycemia and muscular strength in untrained middle-aged patients with type 2 diabetes mellitus. N Am J Med Sci. 2012;4(8):336–43. doi:10.4103/1947-2714.99507. NAJMS-4-336 [pii].
6. Boule NG, Haddad E, Kenny GP, Wells GA, Sigal RJ. Effects of exercise on glycemic control and body mass in type 2 diabetes mellitus: a meta-analysis of controlled clinical trials. JAMA. 2001;286(10):1218–27.
7. Jankowska EA, Wegrzynowska K, Superlak M, Nowakowska K, Lazorczyk M, Biel B, et al. The 12-week progressive quadriceps resistance training improves muscle strength, exercise capacity and quality of life in patients with stable chronic heart failure. Int J Cardiol. 2008;130(1):36–43. doi:10.1016/j.ijcard.2007.07.158.
8. Ades PA, Ballor DL, Ashikaga T, Utton JL, Nair KS. Weight training improves walking endurance in healthy elderly persons. Ann Intern Med. 1996;124(6):568–72.
9. Topp R, Woolley S. Hornyak 3rd J, Khuder S, Kahaleh B. The effect of dynamic versus isometric resistance training on pain and functioning among adults with osteoarthritis of the knee. Arch Phys Med Rehabil. 2002;83(9):1187–95. doi:S0003999302002071 [pii].
10. Winters-Stone KM, Dobek J, Nail L, Bennett JA, Leo MC, Naik A, et al. Strength training stops bone loss and builds muscle in postmenopausal breast cancer survivors: a randomized, controlled trial. Breast Cancer Res Treat. 2011;127(2):447–56. doi:10.1007/s10549-011-1444-z.
11. Singh NA, Clements KM, Fiatarone MA. A randomized controlled trial of progressive resistance training in depressed elders. J Gerontol Ser A Biol Med Sci. 1997;52(1):M27–35.
12. Fiatarone Singh MA, Gates N, Saigal N, Wilson GC, Meiklejohn J, Brodaty H, et al. The Study of Mental and Resistance Training (SMART) Study – resistance training and/or cognitive training in mild cognitive impairment: a randomized, double-blind, double-sham controlled trial. J Am Med Dir Assoc. 2014;15(12):873–80. doi:10.1016/j.jamda.2014.09.010.
13. Daly RM, Dunstan DW, Owen N, Jolley D, Shaw JE, Zimmet PZ. Does high-intensity resistance training maintain bone mass during moderate weight loss in older overweight adults with type 2 diabetes? Osteoporos Int. 2005;16(12):1703–12. doi:10.1007/s00198-005-1906-4.
14. Sigal RJ, Kenny GP, Wasserman DH, Castaneda-Sceppa C. Physical activity/exercise and type 2 diabetes. Diabetes Care. 2004;27(10):2518–39.
15. American College of Sports M, Chodzko-Zajko WJ, Proctor DN, Fiatarone Singh MA, Minson CT, Nigg CR, et al. American College of Sports Medicine position stand. Exercise and physical activity for older adults. Med Sci Sports Exerc. 2009;41(7):1510–30. doi:10.1249/MSS.0b013e3181a0c95c.
16. De Vos NJ, Singh NA, Ross DA, Stavrinos TM, Orr R, Fiatarone Singh MA. Optimal load for increasing muscle power during explosive

resistance training in older adults. J Gerontol A Biol Sci Med Sci. 2005;60(5):638–47.
17. Metter EJ, Conwit R, Tobin J, Fozard JL. Age-associated loss of power and strength in the upper extremities in women and men. J Gerontol Ser A Biol Med Sci. 1997;52(5):B267–76.
18. Miszko TA, Cress ME, Slade JM, Covey CJ, Agrawal SK, Doerr CE. Effect of strength and power training on physical function in community-dwelling older adults. J Gerontol Ser A Biol Med Sci. 2003;58(2):171–5.
19. Von Stengel S, Kemmler W, Kalender WA, Engelke K, Lauber D. Differential effects of strength versus power training on bone mineral density in postmenopausal women: a 2-year longitudinal study. Br J Sports Med. 2007;41(10):649–55. doi:10.1136/bjsm.2006.033480. discussion 55.
20. Bassey EJ, Fiatarone MA, O'Neill EF, Kelly M, Evans WJ, Lipsitz LA. Leg extensor power and functional performance in very old men and women. Clin Sci. 1992;82(3):321–7.
21. Beijersbergen CM, Granacher U, Vandervoort AA, DeVita P, Hortobagyi T. The biomechanical mechanism of how strength and power training improves walking speed in old adults remains unknown. Ageing Res Rev. 2013;12(2):618–27. doi:10.1016/j.arr.2013.03.001.
22. Lexell J, Taylor CC, Sjostrom M. What is the cause of the ageing atrophy? Total number, size and proportion of different fiber types studied in whole vastus lateralis muscle from 15- to 83-year-old men. J Neurol Sci. 1988;84(2–3):275–94.
23. Lexell J. Human aging, muscle mass, and fiber type composition. J Gerontol Ser A Biol Med Sci. 1995;50:11–6.
24. Venojarvi M, Puhke R, Hamalainen H, Marniemi J, Rastas M, Rusko H, et al. Role of skeletal muscle-fibre type in regulation of glucose metabolism in middle-aged subjects with impaired glucose tolerance during a long-term exercise and dietary intervention. Diabetes Obes Metab. 2005;7(6):745–54. doi:10.1111/j.1463-1326.2004.00466.x.
25. Maiorana A, O'Driscoll G, Goodman C, Taylor R, Green D. Combined aerobic and resistance exercise improves glycemic control and fitness in type 2 diabetes. Diabetes Res Clin Pract. 2002;56(2):115–23.
26. Cauza E, Hanusch-Enserer U, Strasser B, Ludvik B, Metz-Schimmerl S, Pacini G, et al. The relative benefits of endurance and strength training on the metabolic factors and muscle function of people with type 2 diabetes mellitus. Arch Phys Med Rehabil. 2005;86(8):1527–33. doi:10.1016/j.apmr.2005.01.007.
27. Nielsen PJ, Hafdahl AR, Conn VS, LeMaster JW, Brown SA. Meta-analysis of the effect of exercise interventions on fitness outcomes among adults with type 1 and type 2 diabetes. Diabetes Res Clin Pract. 2006;74(2):111–20. http://dx.doi.org/10.1016/j.diabres.2006.03.033.
28. Dunbar CC, Robertson RJ, Baun R, Blandin MF, Metz K, Burdett R, et al. The validity of regulating exercise intensity by ratings of perceived exertion. Med Sci Sports Exerc. 1992;24(1):94–9.
29. Day ML, McGuigan MR, Brice G, Foster C. Monitoring exercise intensity during resistance training using the session RPE scale. J Strength Cond Res. 2004;18(2):353–8. doi:10.1519/R-13113.1.
30. Dunstan DW, Daly RM, Owen N, Jolley D, Vulikh E, Shaw J, et al. Home-based resistance training is not sufficient to maintain improved glycemic control following supervised training in older individuals with type 2 diabetes. Diabetes Care. 2005;28(1):3–9.
31. Thiebaud RS, Funk MD, Abe T. Home-based resistance training for older adults: a systematic review. Geriatr Gerontol Int. 2014;14(4):750–7. doi:10.1111/ggi.12326.
32. Lange AK, Vanwanseele B, Foroughi N, Baker MK, Shnier R, Smith RM, et al. Resistive Exercise for Arthritic Cartilage Health (REACH): a randomized double-blind, sham-exercise controlled trial. BMC Geriatr. 2009;9:1. doi:10.1186/1471-2318-9-1.
33. Singh NA, Stavrinos TM, Scarbek Y, Galambos G, Liber C, Fiatarone Singh MA. A randomized controlled trial of high versus low intensity weight training versus general practitioner care for clinical depression in older adults. J Gerontol Ser A Biol Med Sci. 2005;60(6):768–76.
34. Binder EF, Yarasheski KE, Steger-May K, Sinacore DR, Brown M, Schechtman KB, et al. Effects of progressive resistance training on body composition in frail older adults: results of a randomized, controlled trial. J Gerontol Ser A Biol Med Sci. 2005;60(11):1425–31.
35. Simpson KA, Singh MA. Effects of exercise on adiponectin: a systematic review. Obesity. 2008;16(2):241–56. doi:10.1038/oby.2007.53.
36. Pruitt LA, Taaffe DR, Marcus R. Effects of a one-year high-intensity versus low-intensity resistance training program on bone mineral density in older women. J Bone Miner Res. 1995;10(11):1788–95. doi:10.1002/jbmr.5650101123.
37. Perneger TV. What's wrong with Bonferroni adjustments. BMJ. 1998;316(7139):1236–8.
38. Cohen J. Statistical power analysis for the behavioural sciences. New York: Academic Press; 1977.
39. Hozo SP, Djulbegovic B, Hozo I. Estimating the mean and variance from the median, range, and the size of a sample. BMC Med Res Methodol. 2005;5:13. doi:10.1186/1471-2288-5-13.
40. Australian Institute of Health and Welfare. National Health Survey: summary of results, Australia 2007–8. http://www.aihw.gov.au/diabetes/complications/. 2009. Accessed 3 Mar 2015.
41. Australian Institute of Health and Welfare. Australia's health 2014. Australia's health series no. 14. Catalog number AUS 178. Canberra: AIHW; 2014.
42. Look AHEAD Research Group, Bray G, Gregg E, Haffner S, Pi-Sunyer XF, WagenKnecht LE, et al. Baseline characteristics of the randomised cohort from the Look AHEAD (Action for Health in Diabetes) study. Diab Vasc Dis Res. 2006;3:202–15. doi:10.3132/dvdr.2006.031.
43. Hordern MD, Dunstan DW, Prins JB, Baker MK, Singh MA, Coombes JS. Exercise prescription for patients with type 2 diabetes and pre-diabetes: a position statement from Exercise and Sport Science Australia. J Sci Med Sport. 2012;15(1):25–31. doi:10.1016/j.jsams.2011.04.005.
44. LookAHEAD Research Group, Wing RR, Bolin P, Brancati FL, Bray GA, Clark JM, et al. Cardiovascular effects of intensive lifestyle intervention in type 2 diabetes. N Engl J Med. 2013;369(2):145–54. doi:10.1056/NEJMoa1212914.
45. Williamson DA, Rejeski J, Lang W, Van Dorsten B, Fabricatore AN, Toledo K, et al. Impact of a weight management program on health-related quality of life in overweight adults with type 2 diabetes. Arch Intern Med. 2009;169(2):163–71. doi:10.1001/archinternmed.2008.544.
46. Albu JB, Heilbronn LK, Kelley DE, Smith SR, Azuma K, Berk ES, et al. Metabolic changes following a 1-year diet and exercise intervention in patients with type 2 diabetes. Diabetes. 2010;59(3):627–33. doi:10.2337/db09-1239.
47. Lipkin EW, Schwartz AV, Anderson AM, Davis C, Johnson KC, Gregg EW, et al. The Look AHEAD Trial: bone loss at 4-year follow-up in type 2 diabetes. Diabetes Care. 2014;37(10):2822–9. doi:10.2337/dc14-0762.
48. Lee CG, Boyko EJ, Strotmeyer ES, Lewis CE, Cawthon PM, Hoffman AR, et al. Association between insulin resistance and lean mass loss and fat mass gain in older men without diabetes mellitus. J Am Geriatr Soc. 2011;59(7):1217–24. doi:10.1111/j.1532-5415.2011.03472.x.
49. Gianoudis J, Bailey CA, Ebeling PR, Nowson CA, Sanders KM, Hill K, et al. Effects of a targeted multimodal exercise program incorporating high-speed power training on falls and fracture risk factors in older adults: a community-based randomized controlled trial. J Bone Miner Res. 2014;29(1):182–91. doi:10.1002/jbmr.2014.
50. Stengel SV, Kemmler W, Pintag R, Beeskow C, Weineck J, Lauber D, et al. Power training is more effective than strength training for maintaining bone mineral density in postmenopausal women. J Appl Physiol (1985). 2005;99(1):181–8. doi:10.1152/japplphysiol.01260.2004.
51. Orr R, De Vos NJ, Singh NA, Ross DA, Stavrinos TM, Fiatarone-Singh MA. Power training improves balance in healthy older adults. J Gerontol Ser A Biol Med Sci. 2006;61(1):78–85.
52. Ramirez-Campillo R, Castillo A, de la Fuente CI, Campos-Jara C, Andrade DC, Alvarez C, et al. High-speed resistance training is more effective than low-speed resistance training to increase functional capacity and muscle performance in older women. Exp Gerontol. 2014;58C:51–7. doi:10.1016/j.exger.2014.07.001.

Supplementary Table 1. Scores for primary outcomes at all time points for each randomization group

Statistical Model	Time, p-value	Group × Time, p-value	Relative Effect Size (95% CI)
Modified Mini-Mental State Examination			
Model (0, 6, 12)	<0.001	0.06	-0.26 (-0.65, 0.13)
Trail Making Test Part A			
Model (0, 6, 12)	<0.001	0.73	0.10 (-0.28, 0.48)
Trail Making Test Part B			
Model (0, 6, 12)	<0.001	0.06	0.37 (-0.02, 0.77)
Trail Making Test Part B minus Trail Making Test Part A			
Model (0, 6, 12)	0.72	0.08	0.34 (-0.05, 0.73)
Word List Memory			
Model (0, 6, 12)	<0.001	0.55	0.00 (-0.39, 0.39)
Word List Recall			
Model (0, 6, 12)	<0.001	0.07	0.07 (-0.32, 0.45)
Word List Recognition			
Model (0, 6, 12)	0.25	0.35	0.02 (-0.37, 0.42)

Note. CIs: Confidence Intervals. HbA1c: glycosylated hemoglobin. n =103 for all outcomes. All data were normally distributed and raw data used except for Modified Mini-mental State Examination, Trail Making Test Part B minus Trail Making Test Part A, Word list recognition, and Trail Making Test Part B. Trail Making Test Part B data were transformed by taking the inverse before use with parametric statistics. Modified Mini-mental State Examination, Trail Making Test Part B minus Trail Making Test Part A, and Word List Recognition data required log-transformed before use with parametric statistics. An additional separate linear mixed model with repeated measures was used for each cognitive outcome (dependent variable). Intervention fixed effects in mixed models were: Group = Power Training vs. SHAM groups. The additional model was adjusted for age, sex, highest educational level, insulin user, duration of diabetes, HbA1c, Modified Mini-mental State Examination at baseline, and included main effects of time, group, and the interaction term (group × time). P values are from the mixed model Type III Sum of Squares tests of these fixed effects. The relative Hedges' Bias Corrected Effect Sizes (ES) and 95% CIs (confidence intervals) were calculated between the baseline and 12-month timepoints for each cognitive outcome using the estimated marginal means and standard deviations (SD) from the mixed models: Relative effect size (ES) = (Post-test minus Baseline) Power Training - (Post-test minus Baseline) SHAM / pooled baseline SD of cohort. [†]Lower score indicates better function. For all other assessment, higher score indicates better function.

Supplementary Table 2. Associations between changes in cognition and body composition, muscular performance, and cardiorespiratory fitness

Variable	MMMSE		Trails A		Trails B		Trails B minus A		Word list memory		Word list recall		Word list recognition	
	r	p	r	p	r	p	r	p	r	p	r	p	r	p
Skeletal muscle mass														
Skeletal muscle mass (kg)	-0.06	0.57	-0.62	0.09	0.08	0.46	0.11	0.27	0.62	0.09	0.60	0.09	0.34	0.33
Skeletal muscle mass%	-0.53	0.17	0.41	0.28	0.17	0.63	0.04	0.92	0.63	0.04*	-0.10	0.78	0.27	0.46
Thigh muscle CSA (cm^2)	-0.17	0.65	0.60	0.12	-0.06	0.87	-0.26	0.48	0.51	0.17	0.59	0.12	-0.03	0.93
Adiposity														
Visceral fat area cm^2)	0.07	0.85	-0.09	0.84	-0.29	0.47	-0.26	0.52	-0.27	0.41	0.36	0.37	-0.13	0.75
Thigh muscle density	-0.22	0.54	-0.44	0.24	-0.24	0.49	-0.10	0.79	-0.07	0.84	0.32	0.38	0.22	0.54
Body fat mass (kg)	0.07	0.84	0.19	0.61	-0.22	0.53	-0.16	0.65	-0.49	0.11	0.42	0.25	-0.32	0.37
Body fat mass%	0.09	0.87	-0.47	0.21	-0.20	0.58	-0.04	0.89	-0.73	0.02*	0.16	0.66	-0.32	0.36
Waist circumference (cm)	-0.01	0.99	0.12	0.76	0.02	0.96	-0.02	0.96	0.11	0.76	-0.58	0.12	-0.09	0.81
BMI (kg/m^2)	0.13	0.18	0.22	0.55	-0.15	0.66	0.22	0.55	0.09	0.78	0.74	0.12	-0.05	0.89
Muscular performance														
KE maximal strength (Nm)	0.18	0.62	0.64	0.08	-0.51	0.15	-0.93	0.00**	0.37	0.31	0.05	0.89	0.35	0.35
Peak power (w)	-0.54	0.20	0.66	0.12	-0.16	0.70	-0.36	0.39	0.12	0.76	0.23	0.59	0.72	0.10
Cardiorespiratory fitness														
VO_{2peak} (mL/kg/min)	-0.67	0.07	-0.20	0.61	-0.21	0.57	0.14	0.70	-0.15	0.67	-0.52	0.17	-0.61	0.10
VO_{2peak} (L/min)	-0.69	0.06	-0.11	0.78	-0.16	0.64	-0.13	0.71	-0.21	0.53	0.39	0.28	-0.62	0.10

Note. MMMSE: Modified Mini-Mental State Examination; Trails A: Trail Making Test Part A; Trails B: Trail Making Test Part B; Trails B minus A: Trail Making Test Part B minus Trail Making Test Part A; CSA: Cross-sectional Area; BMI, Body Mass Index; KE: Knee Extension; VO2peak: Maximal Aerobic Capacity; r: Standardized Coefficient. Differential group associations between changes in cognition and body composition, cardiorespiratory fitness, and muscular performance were assessed using multiple linear regression models. Each model included change in cognition as the dependent variable (expressed as cognition 12 month – cognition baseline), age, sex, highest educational level, insulin user, and group entered as covariates and a group × independent variable of interest interaction term. *: P<0.05; **: P<0.01; ***: P<0.001.

Supplementary Table 3. Associations between changes in cognition and body composition and muscular performance stratified by group allocation

	Power Training			SHAM		
	r	Coefficient (95% CI)	p-value	r	Coefficient (95% CI)	p-value
Word list memory						
Skeletal Muscle Mass	0.37	0.63 (0.04, 1.26)	0.049*	0.03	0.10 (-0.66, 0.86)	0.79
Fat mass	-0.25	-0.20 (-0.49, 0.09)	0.17	0.15	0.23 (-0.07, 0.53)	0.24
Skeletal Muscle Mass%	0.38	0.66 (0.05, 1.27)	0.03*	-0.15	-0.51 (-1.25, 0.23)	0.23
Fat mass%	-0.40	-0.41 (-0.77, -0.05)	0.02*	0.18	0.37 (-0.07, 0.81)	0.10
Word list recall						
Skeletal Muscle Mass	0.26	0.16 (-0.06, 038)	0.15	-0.07	-0.07 (-0.35, 0.21)	0.62
Trail Making Test Part B minus Trail Making Test Part A						
Knee Extension maximal strength (Nm)	-0.41	-0.14 (-0.26, -0.02)	0.02*	0.19	-0.09 (-0.06, 0.24)	0.24

Note. CIs: Confidence Intervals; SHAM: Sham exercise. Within group associations between changes in cognition and body composition, cardiorespiratory fitness, or muscular performance were assessed using multiple linear regression models, stratified by group allocation. The model included change in cognition as the dependent variable (expressed as cognition 12 month – cognition baseline), and age, sex, highest educational level, and insulin user, and group entered as covariates. Significance represented a significant association between change in cognition and the independent variable of interest within each group, which is presented graphically in Figure 2. Stratification resulted in a significant reduction of the statistical power to determine significance, so the strength of the association (r) was used to inform interpretation in the absence of statistical significance. *: P<0.05; **: P<0.01; ***: P<0.001.

Supplementary Table 4. Associations between changes in cognition and body composition, muscular performance, and cardiorespiratory fitness

Variable	MMMSE		Trails A		Trails B		Trails B minus A		Word list memory		Word list recall		Word list recognition	
	r	p	r	p	r	p	r	p	r	p	r	p	r	p
**Skeletal muscle mass**														
Skeletal muscle mass (kg)	-0.17	0.44	-0.63	0.08	0.25	0.48	0.02	0.96	0.57	0.09	0.59	0.09	0.23	0.51
Skeletal muscle mass%	-0.29	0.23	0.29	0.44	0.12	0.74	0.03	0.94	0.64	0.04*	-0.18	0.64	0.04	0.91
Thigh muscle CSA (cm^2)	-0.02	0.95	0.61	0.13	-0.06	0.88	-0.28	0.46	-0.07	0.84	0.57	0.13	0.56	0.13
**Adiposity**														
Visceral fat area cm^2)	-0.27	0.29	-0.10	0.83	-0.25	0.54	-0.22	0.60	-0.30	0.36	0.36	0.37	-0.15	0.70
Thigh muscle density	-0.19	0.44	-0.48	0.21	-0.15	0.68	-0.01	0.98	0.02	0.96	0.33	0.51	0.09	0.81
Body fat mass (kg)	0.14	0.54	-0.17	0.67	-0.28	0.43	-0.30	0.41	-0.51	0.11	0.50	0.17	-0.22	0.53
Body fat mass%	0.16	0.50	-0.45	0.24	-0.30	0.41	-0.15	0.68	-0.75	0.02*	0.29	0.44	-0.22	0.53
Waist circumference (cm)	-0.05	0.74	0.11	0.78	0.04	0.90	-0.01	0.98	0.06	0.86	0.53	0.14	-0.10	0.79
BMI (kg/m^2)	-0.04	0.86	0.23	0.53	-0.13	0.59	-0.21	0.54	0.07	0.84	0.54	0.11	0.07	0.84
**Muscular performance**														
KE maximal strength (Nm)	0.26	0.28	0.61	0.10	-0.50	0.17	-0.98	0.01**	0.34	0.31	0.12	0.73	0.28	0.44
Peak power (w)	0.07	0.80	0.69	0.11	-0.39	0.36	-0.18	0.67	0.13	0.76	0.23	0.60	0.79	0.06
**Cardiorespiratory fitness**														
VO_{2peak} (mL/kg/min)	-0.14	0.56	-0.21	0.60	-0.26	0.49	-0.19	0.61	-0.11	0.76	-0.46	0.22	-0.64	0.08
VO_{2peak} (L/min)	-0.27	0.22	-0.11	0.78	-0.21	0.56	-0.17	0.62	-0.18	0.59	-0.34	0.35	-0.61	0.08

Note. MMMSE: Modified Mini-Mental State Examination; Trails A: Trail Making Test Part A; Trails B: Trail Making Test Part B; Trails B minus A: Trail Making Test Part B minus Trail Making Test Part A; CSA: Cross-sectional Area; BMI, Body Mass Index; KE: Knee Extension; VO_{2peak}: Maximal Aerobic Capacity; r: Standardized Coefficient. HbA1c: glycosylated hemoglobin. Differential group associations between changes in cognition and body composition, cardiorespiratory fitness, and muscular performance were assessed using multiple linear regression models. Each additional model included change in cognition as the dependent variable (expressed as cognition 12 month – cognition baseline), age, sex, education, insulin user, HbA1c, duration of diabetes, and MMMSE at baseline and group entered as covariates and a group × independent variable of interest interaction term. *: P<0.05; **: P<0.01; ***: P<0.001.

Supplementary Table 5. Associations between changes in cognition and body composition and muscular performance stratified by group allocation

	Power Training			SHAM		
	r	Coefficient (95% CI)	p-value	r	Coefficient (95% CI)	p-value
Word list memory						
Skeletal Muscle Mass	0.39	0.67 (0.02, 1.36)	0.046*	0.02	0.05 (-0.75, 0.86)	0.89
Fat mass	-0.27	-0.21 (-0.52, 0.09)	0.16	0.15	0.23 (-0.07, 0.53)	0.24
Skeletal Muscle Mass%	0.39	0.66 (0.05, 1.27)	0.04*	-0.18	-0.51 (-1.25, 0.23)	0.12
Fat mass%	-0.40	-0.42 (-0.79, -0.04)	0.03*	0.21	0.42 (-0.04, 0.88)	0.08
Word list recall						
Skeletal Muscle Mass	0.15	0.16 (-0.09, 039)	0.21	-0.03	-0.03 (-0.33, 0.27)	0.82
Trail Making Test Part B minus Trail Making Test Part A						
Knee Extension maximal strength (Nm)	-0.43	-0.15 (-0.26, -0.03)	0.01*	0.19	-0.09 (-0.07, 0.24)	0.27

Note. CIs: Confidence Intervals; SHAM: Sham exercise; HbA1c: glycosylated hemoglobin. Within group associations between changes in cognition and body composition, cardiorespiratory fitness, or muscular performance were assessed using multiple linear regression models, stratified by group allocation. Each additional model included change in cognition as the dependent variable (expressed as cognition 12 month – cognition baseline), and age, sex, insulin usage, HbA1c, and duration of diabetes at baseline and group entered as covariates. Significance represented a significant association between change in cognition and the independent variable of interest within each group. Stratification resulted in a significant reduction of the statistical power to determine significance, so the strength of the association (r) was used to inform interpretation in the absence of statistical significance. *: P<0.05; **: P<0.01; ***: P<0.001.
